# Utilization of Eggshell Waste Calcite as a Sorbent for Rare Earth Element Recovery

**DOI:** 10.1021/acsomega.4c00931

**Published:** 2024-06-04

**Authors:** Rémi Rateau, Melanie Maddin, Adrienn M. Szucs, Luca Terribili, Kerstin Drost, Paul C. Guyett, Juan Diego Rodriguez-Blanco

**Affiliations:** † Discipline of Geology, School of Natural Sciences, Trinity College Dublin, the University of Dublin, Museum Building, College Green, Dublin D02 PN40, Ireland; ‡ iCRAG, the Science Foundation Ireland Research Centre in Applied Geosciences, O’Brien Centre for Science (East), Dublin D02, Ireland

## Abstract

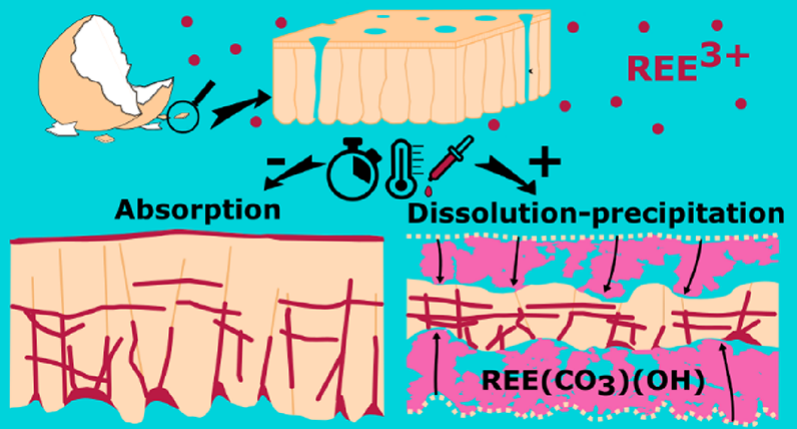

The green energy transition requires rare earth elements (REE) for the permanent magnets used in electric cars and wind turbines. REE extraction and beneficiation are chemically intensive and highly damaging to the environment. We investigated the use of eggshell waste as a sustainable alternative sorbent for the capture and separation of REE from aqueous solutions. Hen eggshell calcite was placed in multi-REE (La, Nd, Dy) solutions at 25 to 205 °C for up to 3 months. A pervasive diffusion of the REE inside the eggshell calcite was observed along pathways formed by the intracrystalline organic matrix and calcite crystal boundaries. At 90 °C, kozoite (REECO_3_OH, orthorhombic) spherulites precipitate on the surface of the dissolving calcite. At 165 and 205 °C, an interface-coupled dissolution–precipitation mechanism is observed, resulting in the complete dissolution of the calcite shell and its pseudomorphic replacement by polycrystalline kozoite. At 205 °C, kozoite is slowly replaced by hydroxylbastnäsite (REECO_3_OH, hexagonal), the stable form of the rare earth hydroxycarbonate polymorphs. Our results demonstrate two potential applications of eggshell waste for the uptake of rare earth elements in solution: at low temperatures, as a mixed organic–inorganic adsorbent and absorbent, given sufficient sorption time; and at higher temperatures, as an efficient sacrificial template for the precipitation of rare earth hydroxycarbonates.

## 1 Introduction

Wind electricity and electric vehicles are two key pillars of the green energy transition,^1^ one of the main strategies to reach net zero CO_2_ emission by 2050.^2^ Both technologies depend upon the NdFeB permanent magnet technology.^3^ These magnets typically contain 28–35 wt %^4^ of rare earth elements (REE); mostly Nd (20–30 wt %), with up to a few wt % each of Pr, Dy, and Tb.^5,6^

Historically, few countries have been willing to operate REE mines due to their high environmental and health risk, caused by the release of large quantities of toxic and mildly radioactive sludges.^7^ However, as the worldwide production of REE must significantly increase to meet the energy transition demand,^8^ more countries are now open to REE mining and processing.^9,10^

To reduce the environmental footprint of REE production, there has been an increased interest toward recovery from recycling REE-rich waste materials^11^ and toward the use of waste materials to extract REE from aqueous solutions in place of more damaging chemical processes.^12^ One such tested waste material is a hen eggshell. The food industry produces a large amount of eggshell waste. In 2021, an estimated 1.7 trillion eggs were produced worldwide (see calculations in the Supporting Information (SI), Text S1).^13^ Given an average mass of six grams^14^ and 93.5% calcite,^15^ an estimated 10 million metric tons of eggshell calcite is produced annually, of which a large portion is disposed of in landfills,^16^ where their bacterial and viral content might become a health risk.^17^

Since the beginning of the century,^18^ eggshells have been tested as a potential low cost sorbent for heavy metal removal from waste waters,^19^ but only a handful of studies have focused on the recovery of REE. The adsorption capacity of eggshells was found to be 100 mg·g^–1^ for La,^20^ and 19^21^ or 163 mg·g^–1^^22^ for Eu, depending on experimental conditions. Eggshells were also used to recover ca. 90% of both Nd and Ce from a SEDEX-type ore leachate.^23^ The uptake of REE by eggshells in these studies has been mechanistically attributed to either adsorption^20−22^ or ion exchange.^23^ There has been no study on precipitation as a mechanism for REE capture, as occurring with inorganic calcite and rare earth carbonates in aqueous solutions with REE.^24−28^

In this study, we aim to investigate the capacity of eggshell calcite to capture and partition REE from high concentration multi-REE aqueous solutions. We focus on the characterization of the mineralogy, crystallization pathways, structural features, and elemental distribution of the products of the reaction between eggshells and multi-REE solutions. By contrasting the results with those obtained from inorganic calcite crystals, we aim to qualify the potential of eggshell waste to act as a reactant for the sustainable recovery of REE.

## 2 Materials and Methods

Commercial hen eggshells were cleaned with soap and water, air-dried, crushed in a ceramic mortar, and sieved. The 1–2 mm fraction was treated in a 1 M NaOH solution for 24 h and then washed with deionized water and air-dried. Iceland spar calcite crystals were also crushed and sieved to obtain separate 1–2 mm grains. Three single REE bearing solutions were produced with a REE concentration of 50 mM. We selected La, Nd, and Dy as (1) they are representative of both the light (La, Nd) and heavy (Dy) REE; (2) the La to Dy ionic radii range represents 72% of the ionic radii range of the lanthanides; (3) La and Nd are the two most abundant REE in the crust after Ce, and Dy is the most abundant heavy REE;^29^ and (4) Nd and Dy are the most demanded REE for the energy transition.^30^ The solutions were produced by dissolving single REE nitrate salts, REE(NO_3_)_3_·6H_2_O (Sigma-Aldrich, 99.9% purity), in deionized water (18.18 MΩ·cm). For each experiment, 0.05 g of eggshells was placed in a 20 mL Teflon reactor with 5 mL of each of the three single REE solutions to obtain 15 mL of a 50 mM multi-REE solution (with La, Nd, Dy; ca. 16.67 mM each). Each reactor was capped with a Teflon lid and placed in a stainless steel autoclave to keep the system closed during the experiment. Narrow-mouth Nalgene polypropylene bottles (Thermo Fisher Scientific) were used as reactors for the ≤90 °C experiments. The reactors were placed in the oven at temperatures of 30, 90, 165, or 205 °C from 3 h to 3 months. Some experiments were also run at room temperature, ca. 20 °C. Experiments run at room temperature and 30 °C will be reported as a single set of experiments associated with a temperature of 25 (±5) °C. Once the experiments were completed, the grains were manually retrieved with a metallic spatula, placed in plastic Eppendorf tubes, and dried in an oven at 40 °C for 24–48 h. For mineral identification and quantification, a few grains of each experiment were randomly selected and crushed by using an agate mortar and pestle. The powder was analyzed using a Siemens/Bruker D5000 power X-ray diffractometer (Cu Kα radiation, 0.01° step^–1^ from 5 to 60° 2θ at 0.2° min^–1^, 4.5 h scan per sample). Mineral identification was undertaken with DIFFRAC.EVA (Bruker) using the Powder Data File (PDF-4, The International Centre for Diffraction Data).^31^ The quantification of crystalline phases was carried out using the Rietveld refinement program TOPAS (Bruker).^32^

For structural imaging and elemental mapping, scanning electron microscopy (SEM) analyses were carried out at the iCRAG Lab@TCD at Trinity College Dublin (Ireland) on a Tescan TIGER MIRA3 FEG-SEM equipped with two Oxford Instruments Ultim Max 170 mm^2^ SSD/EDX detectors and an X4 pulse processor. Scanning electron (SE) and backscattered electron (BSE) imaging and EDS analyses of the carbon-coated pin stubs and pucks (see SI Text S2 for a description of the stub and puck preparation) were acquired using an accelerating voltage of 20 keV and a working distance of 15 mm. High-resolution SE imaging of gold-coated stubs were acquired using an accelerating voltage of 5 keV and a working distance of 5 mm. The images and maps were processed by using the AZtec v6.1 X-ray microanalysis software suite (Oxford Instruments). For quantitative elemental spectrum analyses and mapping, the primary standard was Micro-Analysis Consultants registered standard no. 2808 (Agar Scientific). The secondary standard was a monazite from MINM25–53 (serial 1CT) standard (Astimex Standards Ltd.). The results of the secondary standard analysis for the three REE are presented in SI Table S1 and indicate an uncertainty for the unknowns of ±4%.

For trace element mapping in calcite, LA-ICP-Q-MS analyses were carried out on an Agilent 7900 Q-ICP-MS coupled to an Iridia 193 nm ArF excimer laser ablation system (Teledyne Photon Machines, Bozeman, MT, USA). Maps were acquired by ablating successive linear rasters using a fluence of 2.5 J·cm^2^, a repetition rate of 50 Hz, a 10 μm square spot, and a scan speed of 15 μm·s^–1^ (*i.e*., the sample stage moved along a linear array beneath the stationary laser beam by 15 μm·s^–1^). The rasters were set up perpendicular to the outer and inner rims of the eggshell cross sections. NIST SRM 610 glass was used as primary trace element concentration standard.^33^ The following masses were measured: major element ^43^Ca (0.01 s integration time), minor elements ^24^ Mg (0.01 s), ^31^P (0.02 s), ^57^Fe (0.01 s), and ^88^Sr (0.01 s), and for the three REE ^139^La (0.02 s), ^145^Nd (0.03 s), ^146^Nd (0.03 s), and ^163^Dy (0.04 s), with a total integration time of 0.2 s. The data was acquired using Chromium v3.1 (Teledyne Photon Machines) for the laser ablation and MassHunter v4.3 (Agilent Technologies) for the mass spectrometry. The data was processed using Iolite v4, a data processing software package for time-resolved mass spectrometry data.^34,35^

Chemical species speciation, mineral saturation indexes, and the kinetics of dissolution of calcite, for the range of experimental conditions, were calculated using the USGS aqueous geochemical modeling program PHREEQC Interactive v3.7.^36^ We used the built-in llnl.dat, adapted from the thermo.com.V8.R6.230 database from the Lawrence Livermore National Laboratory,^37^ as the thermodynamic database. The database was supplemented by the addition of two phases, kozoite-(Nd) and hydroxylbastnäsite-(Nd), used as representative proxies of the phases that we found in our reaction products. The solubility product constants for both phases are extracted from Voigt et al. (see rationales in SI Text S3 and Table S2).^38^ The enthalpies of reaction of both minerals, required to calculate their solubility at temperatures other than 25 °C with the Van’t Hoff equation,^36^ were derived from the enthalpies of formation of the reactant and products of the dissolution reaction by using Hess’s Law (see SI Text S3 and Table S3 for a derivation of the values). Calcite dissolution was modeled using the llnl.dat built-in kinetics model.^39,40^ The overall modeling strategy is explained in SI Text S4, and a copy of the PHREEQC input file is presented in SI Text S5.

## 3 Results and Discussions

### 3.1 Mineralogy and Crystallization Pathway

**Figure 1:**
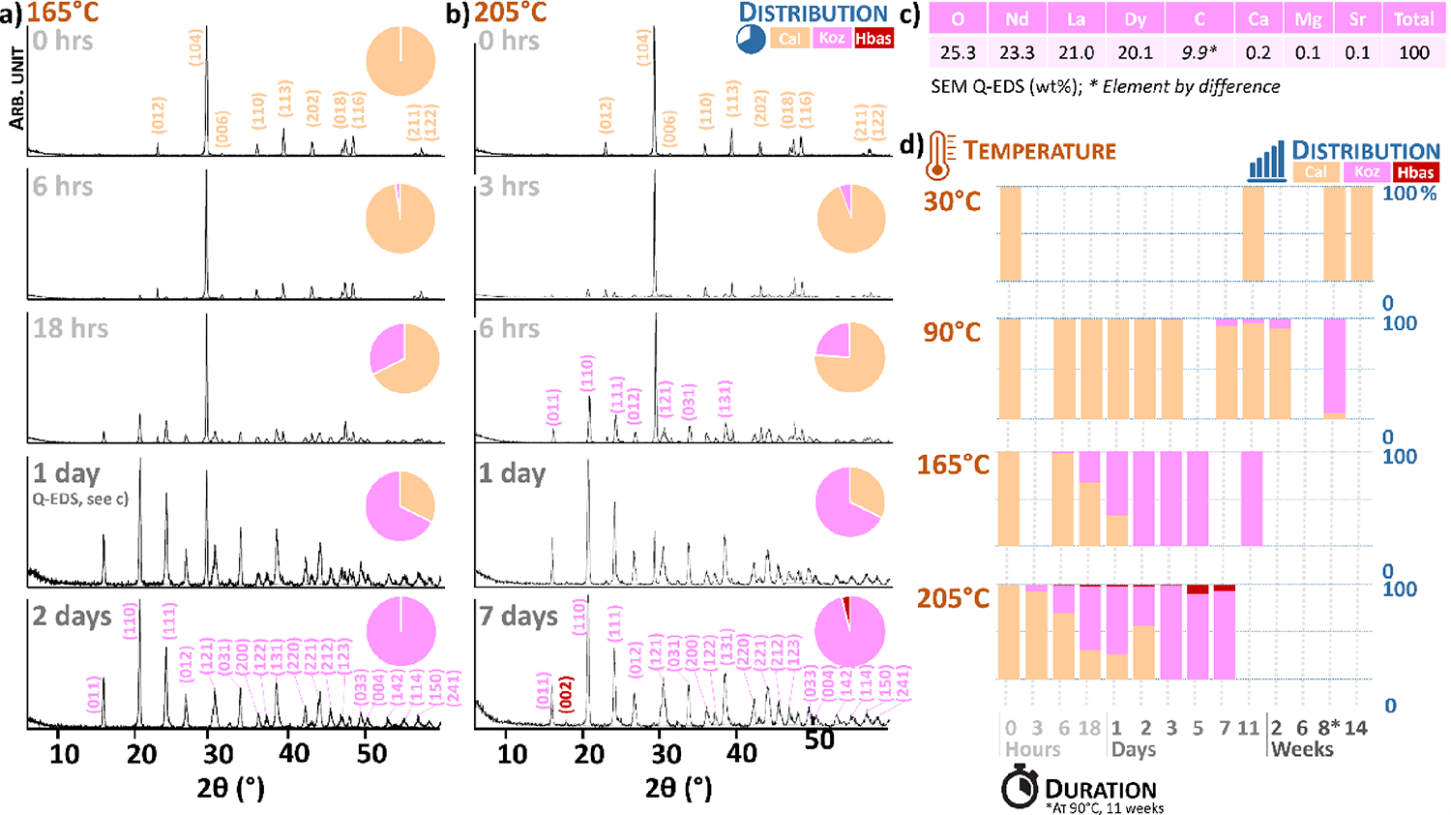
Mineralogical and elemental compositions of the experiment products. (a) XRD patterns and quantitative mineralogical analyses of selected experiments with eggshells at 165 °C; (b) at 205 °C; (c) kozoite major and minor elements (wt %), as represented by a selected kozoite crystal after 1 day at 165 °C (standard-based quantitative analysis, with C, the conductive coating element, calculated by difference between the sum of the other elements and 100 wt %); (d) quantitative phase analyses for all experiments. Arb. unit = arbitrary unit; Cal = calcite; Koz = kozoite; Hbas = hydroxylbastnäsite; Q-EDS = quantitative EDS.

**Figure 2:**
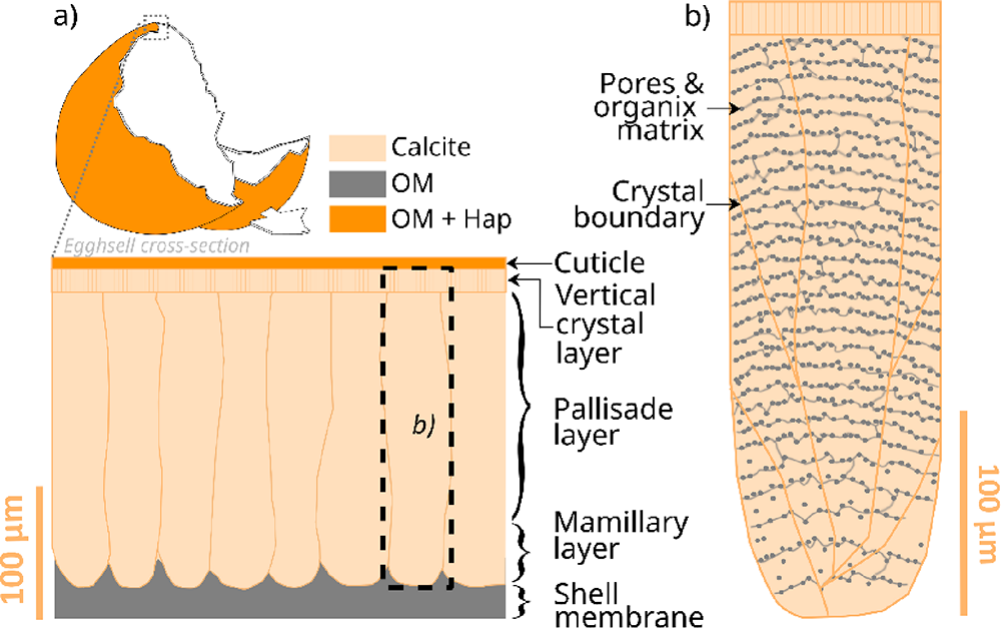
Structure of the hen eggshell. (a) Schematic structure of the eggshell; (b) internal structure of the eggshell calcite. Legend: Hap = hydroxyapatite; OM = organic matter.

The XRD pattern of unreacted eggshells revealed the presence of calcite (ICDD PDF no. 00–047–1743) (Figure 1a), although minor amounts of vaterite and hydroxyapatite have been reported within the basal part of the palisade layer and the cuticle (Figure 2a).^41^

The XRD patterns of the reacted grains were matched to three phases: calcite, and two rare earth carbonate polymorphs with formula REE(CO_3_)(OH), namely, kozoite^42^ (orthorhombic, ICDD PDF no. 04–014–4501) and hydroxylbastnäsite^43^ (hexagonal, ICDD PDF no. 00–038–0400) (Figure 1a,b). Kozoite is a member of the ancylite mineral group, (REE,Ca, Sr)_2_(CO_3_)_2_(OH,H_2_O)_2_, together with the isostructural calcioancylite, (REE,Ca)_2_(CO_3_)_2_(OH,H_2_O)_2_,^44^ but the absence of Ca in the precipitated phase (≪0.1%, Figure 1c) rules out the phase calcioancylite. SEM-EDS mapping revealed that the two hydroxycarbonate polymorphs incorporated all three REE (Figure 1c) and are, therefore, solid solutions with the generic formula (La_*x*_,Nd_*y*_,Dy_1–*x*–*y*_)(CO_3_)(OH).

With the eggshells, kozoite was detected in minute quantity at 90 °C after 1 day, amounting to a few weight percent after 2 weeks and close to 100% after 80 days, while at 165 °C, calcite was gradually replaced by kozoite, and after 2 days, only kozoite remained (Figure 1d). The same replacement pattern was observed at 205 °C; however, additionally, there was emergence followed by a slow but gradual increase of hydroxylbastnäsite (Figure 1d), suggesting that it replaces the previously precipitated kozoite. Therefore, the evident crystallization pathway of our system is calcite → kozoite → hydroxylbastnäsite. This crystallization pathway has previously been reported for inorganic Iceland spar calcite crystals and single REE solutions over the same temperature range.^24,25^ These studies identified a lanthanite precipitation step, REE_2_(CO_3_)_3_·8H_2_O, before the formation of kozoite but only for experiments with light REE solutions, at the low temperature of 21 °C,^24^ and in low quantities due to slow growth kinetics.^25^ The absence of lanthanite in our 25 °C experiments could therefore be caused by the combination of the presence of Dy in the solution and the lack of data for reaction times greater than 100 days.

At 25 °C, the retrograde solubility of calcite, *K*_SP-cal_, is 10^–8.48^,^45^ for the reaction Ca(CO_3_) ⇌ Ca^2+^ + CO_3_^2–^, and decreases to ca. 10^–11.5^ at 205 °C.^46^ For our synthetic multi-REE polymorphs, we will use kozoite-(Nd) and hydroxylbastnäsite-(Nd) as thermodynamic proxies (see the rationale in SI Text S3). At 25 °C, the solubility product constants of kozoite, *K*_SP-koz-(Nd)_, and hydroxylbastnäsite, *K*_SP-hbas-(Nd)_, are 10^–22.3^ and 10^–23.8^, respectively.^38^ As temperature increases, kozoite and hydroxylbastnäsite display prograde solubilities, with *K*_SP_ increasing to 10^–12.8^ and 10^–22.9^ at 205 °C, respectively (SI Text S3).^38^ Consequently, at the range of temperatures used in this study, calcite is always significantly more soluble than the rare earth hydroxycarbonate polymorphs, and kozoite is always more soluble than hydroxylbastnäsite, particularly as temperature increases, with the difference reaching 10 orders of magnitude at 205 °C. As hydroxylbastnäsite is the less soluble of the two polymorphs, at these temperatures, kozoite can be considered as metastable and hydroxylbastnäsite as stable. The observed crystallization pathway, starting with the less stable polymorph, kozoite, follows the commonly observed Ostwald’s Rule of Stages.

### 3.2 Eggshell Calcite Resistance to Dissolution

Geochemical modeling indicates that the starting solution, in equilibrium with atmospheric air, has a pH of 5 at 25 °C, decreasing to 3.3 at 205 °C, and an ionic strength within the range of 0.25–0.30 mol·kg_water_^–1^ (SI Figure S2). The thermodynamic and kinetics modeling of eggshell calcite dissolution in the presence of the starting solution indicates that at 25 °C, the solution should reach saturation with respect to calcite after ca. 100 days and after the dissolution of almost the entirety of the 500 μmol of calcite SI Figure S2). Contrary to the model prediction, our experiments at 25 °C do not show any visible dissolution of calcite even after 98 days (SI Figure S3), suggesting that the dissolution kinetics of the hen eggshell calcite might differ significantly from the spar calcite used to empirically quantify the dissolution model^39^ and implying that eggshell calcite is more stable in acid aqueous solutions than inorganic calcite. The inhibition and slowing down of calcite dissolution could be partly caused by the medium-high ionic strength of the solution.^47^ Enhanced stability, when compared to inorganic calcite, has also been observed in other biogenic calcites, such as in coccoliths, with the inhibitor believed to be an organic coating.^48^ The complex organic matrix permeating the entire hen eggshell^41^ might therefore be also partly responsible for the eggshell resistance to dissolution at low temperatures.

### 3.3 Calcite → Kozoite → Hydroxylbastnäsite Pseudomorphic Interface Coupled Dissolution–Precipitation

**Figure 3:**
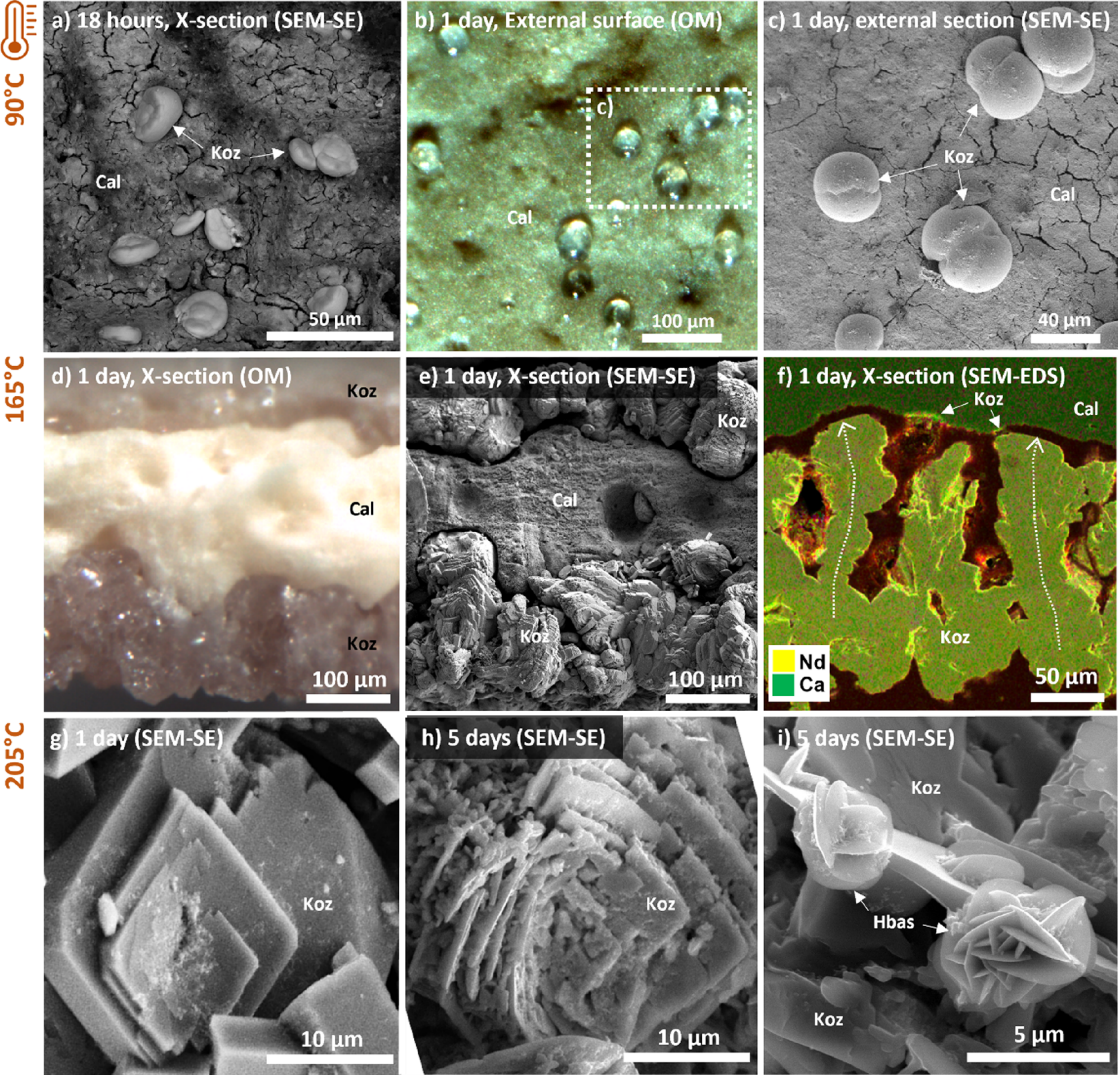
Optical and SEM imaging of the experiment products at (a–c) 90, (d–f) 165, and (g–i) 205 °C. (a) 18 h: kozoite spherulites; (b) transparent colorless spherulite growing on the external surface of the shell; (c) SEM-SE close-up of (b); (d) 1 day: two centripetal replacement fronts; (e) same as (f) but with SEM-SE; (f) kozoite columnar growth, replacement front gap, and kozoite growing on the dissolving calcite core; (g) 1 day: prismatic kozoite; (h) after 5 days: dissolution pits. (i) after 5 days: growth of “desert-rose”-shaped hydroxylbastnäsite on the dissolving kozoite. OM = optical microscopy.

At 90 °C, a minute quantity of kozoite, the metastable precursor to hydroxylbastnäsite, appeared after 1 day and reached 94% after 80 days (Figure 1d). The crystals displayed spherulitic morphologies, either discoidal (Figure 3a) or spherical (Figure 3b,c), often composites of multiple merged spherulites; and colorless to slightly pinkish and transparent (Figure 3b) to vitreous, reminiscent of some of the natural kozoite crystals found in Japanese basalts.^49^ The pink color is due to Nd in the kozoite lattice.^42,49^

At 165 °C, we observed a complete replacement of calcite by kozoite after 2 days (Figure 1a,d) and a preservation of the volume and shape of the original eggshell grains (SI Figure S3). Two replacement fronts, parallel to the surface of the eggshells, were observed progressing from the external and internal surfaces toward the inner core (SI Figure S3, Figure 3d,e). After 2 days, these centripetal replacement fronts met, and only kozoite remained. SEM images revealed a polycrystalline structure with preferential longitudinal growth of kozoite and a homogeneous crystal size distribution with average lengths of several tens of micrometers and reaching up to 80 μm, and the presence of significant porosity between the main columnar clusters of kozoite (Figure 3e,f). The replacement front is characterized by a several micrometer-wide gap between the growing kozoite and the dissolving calcite. Preferential growth of kozoite was observed on the surface of kozoite in direct contact with the replacement front gap, while only a minor amount of kozoite was observed crystallizing on the surface of the calcite (Figure 3f).

The thermodynamical modeling shows that the bulk solution is undersaturated with respect to kozoite, even once the solution has reached saturation with respect to calcite (SI Figure S2). For kozoite to precipitate in an undersaturated solution, it is necessary for oversaturation to occur within a subset of the bulk solution. Such local oversaturation can be created during interface coupled dissolution–precipitation reactions, observed in many aqueous-based mineral replacement systems.^50−52^ In the case of our calcite–kozoite system, the dissolution of a few monolayers of the substrate mineral releases both Ca^2+^ and CO_3_^2–^ ions in the solution water, which, if the diffusion rate in our nonstirred water is slower than the dissolution rate, creates a concentration gradient in the water layer immediately in contact with the dissolving calcite, with a rapid decrease in concentration away from the water-calcite interface. This water layer with a high concentration of dissolved ions is termed the boundary layer. It rapidly becomes oversaturated with respect to one of the insoluble REE hydroxycarbonate polymorphs, which precipitate on the surface of the substrate. The dissolution and precipitation rates become coupled, leading in some circumstance to complete pseudomorphic replacement of the substrate phase by a less soluble phase,^50^ in our case, kozoite.

While the individual kozoite crystals in our experiments are not pseudomorphs of the original palisade calcite crystals (compare Figures 2b and 3f), the preservation of the overall volume and shape of the eggshell grains by polycrystalline kozoite can be described as a polycrystalline pseudomorphic replacement. This type of pseudomorphism has been suggested to be the consequence of a low degree of epitaxy, or structural match, between the dissolving and precipitating phase.^51^ While epitaxic relationships have been observed between kozoite and calcite,^24^ the polycrystalline nature of eggshell calcite, combined with the presence of a pervasive intracrystalline organic matric, might result in a lower degree of epitaxy than with inorganic spar calcite. We note that full replacement occurs despite the difference in molar volume between calcite, 36.9 cm^3^.mol^–1^, and kozoite, 46.4 cm^3^.mol^–1^, resulting in a 25% increase in volume of the precipitate when compared to the molar equivalent of dissolved substrate. Therefore, there must be a significant quantity of carbonate ions not bonding with REE ions near the grain surface but instead leaving the boundary layer and remaining as dissolved aqueous ions in the bulk solution.

Interface coupled dissolution–precipitation is also observed in our experiments with inorganic spar calcite and has been reported in previous studies.^24,25^ However, at 165 °C, even after 7 days, more than 60% of calcite remains (Figure 1d). The kozoite crystals are also smaller in size than those with eggshell calcite. After a few days, the armoring of the calcite creates a water-tight kozoite crust that isolates the remnant calcite core from the rest of the bulk solution. The water trapped between the calcite core and the impermeable kozoite crust quickly reaches partial equilibrium^53^ with respect to calcite and kozoite, and the dissolution stops. When compared to eggshell calcite, the lack of permeability in the kozoite crust of the inorganic calcite is possibly due to the growth of significantly smaller kozoite crystals, allowing for a tighter crystal arrangement as well as a quicker precipitation rate.

At 205 °C with eggshell calcite grains, a similar polymorphic replacement is observed, occurring within the same time scale. However, while kozoite is the dominant replacement phase after 1 day, a minor amount of hydroxylbastnäsite has also precipitated (Figure 1b,d). The kozoite crystals are at this stage prismatic and have very few defects (Figure 3g). After 5 days, the kozoite crystals show numerous dissolution pits and are sparingly covered with “desert rose”-shaped crystals (Figure 3h,i), interpreted as the hydroxylbastnäsite identified in the XRD data, demonstrating that hydroxylbastnäsite replaces kozoite. The appearance of the stable polymorph of the REE hydroxycarbonate at 205 °C is probably due to the combined effect of temperature: (1) on accelerating the otherwise slow growth kinetics of hydroxylbastnäsite, and (2) on the increased solubility of kozoite, which is now 10 orders of magnitude higher than hydroxylbastnäsite. Given enough time and given the slightly smaller molar volume of hydroxylbastnäsite, i.e., 45.2 cm^3^·mol^–1^, we expect kozoite to be entirely replaced by hydroxylbastnäsite. Previous studies have shown that, at 220 °C, kozoite-(La), kozoite-(Pr), and kozoite-(Nd) are fully replaced by their hydroxylbastnäsite counterparts within 7 days or less, whether kozoite has been homogeneously precipitated^54^ or results from the dissolution–precipitation of calcite^24^ or dolomite.^25^ Contrary to the experiments with inorganic calcite and with single REE solutions, less than 4% of kozoite has been replaced by hydroxylbastnäsite in the eggshell multi-REE experiments at 205 °C after 7 days (Figure 1), suggesting an inhibition and delay of the polymorphic transformation. Therefore, we suggest that in the case of eggshells and a multi-REE solution containing Dy, the full replacement of kozoite by hydroxylbastnäsite might take several weeks or months to be completed.

### 3.4 Absorption of REE by Eggshell Calcite

**Figure 4:**
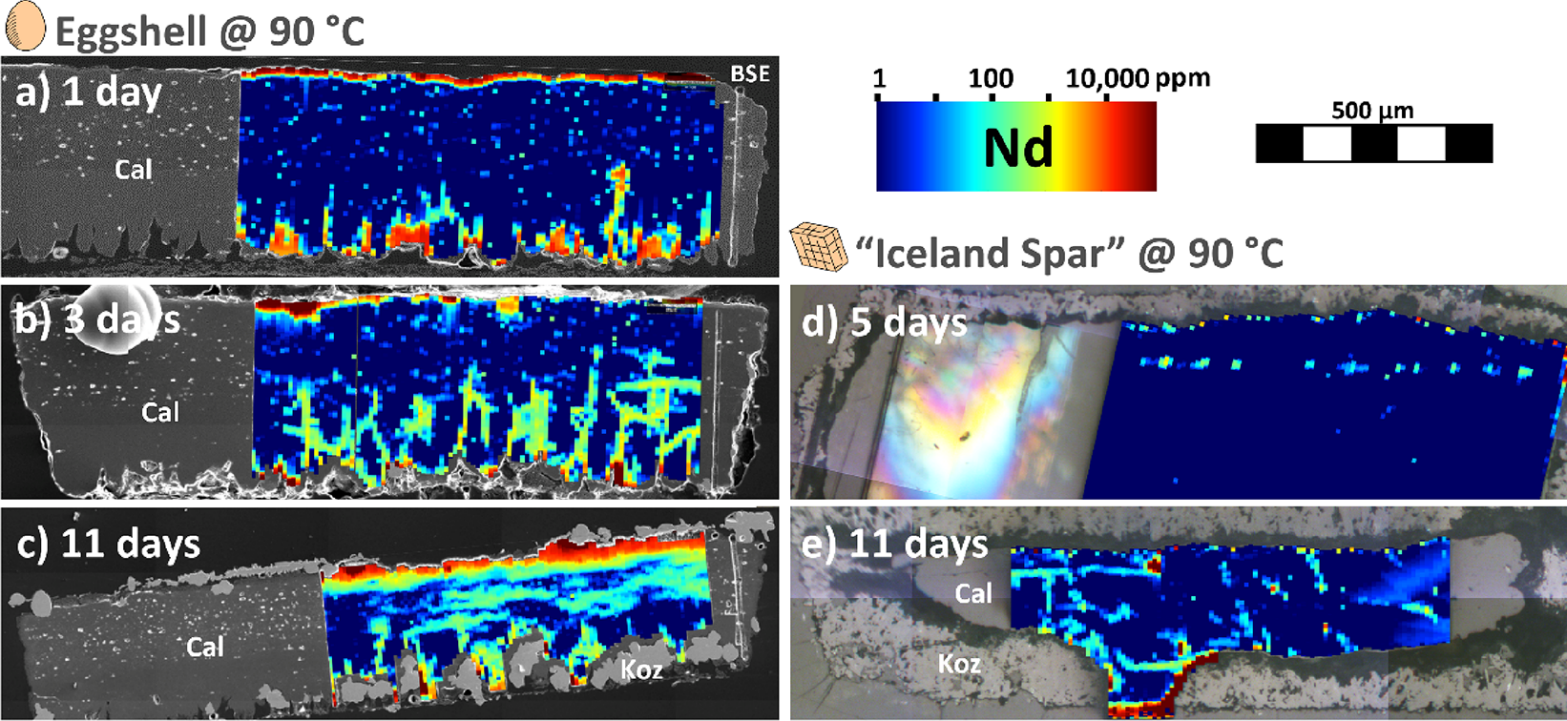
Nd concentration and spatial distribution within the eggshell calcite at 90 °C, after: (a) 1 day; (b) 3 days; (c) 11 days; and within the Iceland spar calcite, after: (d) 5 days; (e) 11 days.

LA-ICP-MS maps of the calcite after 24 h at 90 °C showed up to several tens of thousands of ppm of REE within the first 50 μm of the external surface, at the intersections between mammillae, as vertical features extending from the internal surface toward the core; and as dots spread evenly throughout the calcite grain (Figure 4a). After 3 days, the internal section of the eggshell showed the growth of the vertical features, which are now preponderant; after 11 days, distinct long horizontal features became visible within the calcite core, which previously exhibited only isolated dots (Figure 4b,c).

This pattern of high REE concentration can be related to unique features of eggshell calcite: (1) the narrow external zone corresponds to the vertical crystal layer; (2) the vertical linear features between the mammillae likely correspond to calcite grain boundaries; and (3) the dots and horizontal lines in the palisade layer are probably related to the horizontally aligned vacuoles and connecting flocculent organic matter matrix^41^ (Figure 2b).

When comparing with inorganic calcite, after 5 and 11 days at 90 °C, we observe very little diffusion of REE within the calcite grain (Figure 4d,e), with only a few linear or curvilinear REE-rich features (on the order of 100 ppm at the maximum), interpreted as cleavage planes and microfractures.

As the closure temperature for diffusion of Nd in 100 μm calcite grains is close to 500 °C,^55^ volume diffusion of REE in the actual crystallographic matrix of calcite is expected to be insignificant at our experimental temperatures, as is confirmed by our spar calcite experiments. Thus, we interpret the observed diffusion of REE in eggshell calcite as occurring along the calcite grain boundaries and along the intracrystalline web of organic matter.

Compared to spar calcite, the high REE absorption capacity of eggshell calcite would result in a localized lower concentration of REE ions in the fluid boundary layer responsible for the interface coupled dissolution–precipitation reaction. This localized lower concentration might result in slower REE carbonate precipitation kinetics. On the contrary, the low REE absorption capacity of inorganic spar calcite, combined with a higher dissolution rate, would favor a higher kozoite precipitation rate, resulting in the observed smaller crystal size and the resulting higher likelihood of developing a water-tight armoring.

The absorption of REE by eggshell calcite occurs even at our lowest tested temperature of 30 °C, where the REE diffusion pattern is after 95 days (SI Figure S4) resembles the diffusion pattern after 1 day at 90 °C (Figure 4a). While slow, the REE absorption capacity of eggshell calcite at close to room temperatures could be considered as a low environmental footprint sorption mechanism on its own, with no heating requirement.

Previous adsorption studies with eggshell calcite, with contact time of up to 24 h, always interpreted the sorbed REE as having been adsorbed on the eggshell surface.^20−22^ Our data proves that REE are not only adsorbed as mono- or multiionic layers on the calcite surface, a fundamental assumption of the adsorption models, but are also absorbed within the organic matrix and along crystal boundaries. Although LA-ICP-MS data for reaction times ≤24 h, equivalent to typical contact times in adsorption studies, are not available, we propose that future investigations should explore the impact of absorption on their findings. The extent of absorption within this time frame could challenge the validity of their model assumptions and consequently affect the quantitative estimates of adsorption capacity.

**Figure 5:**
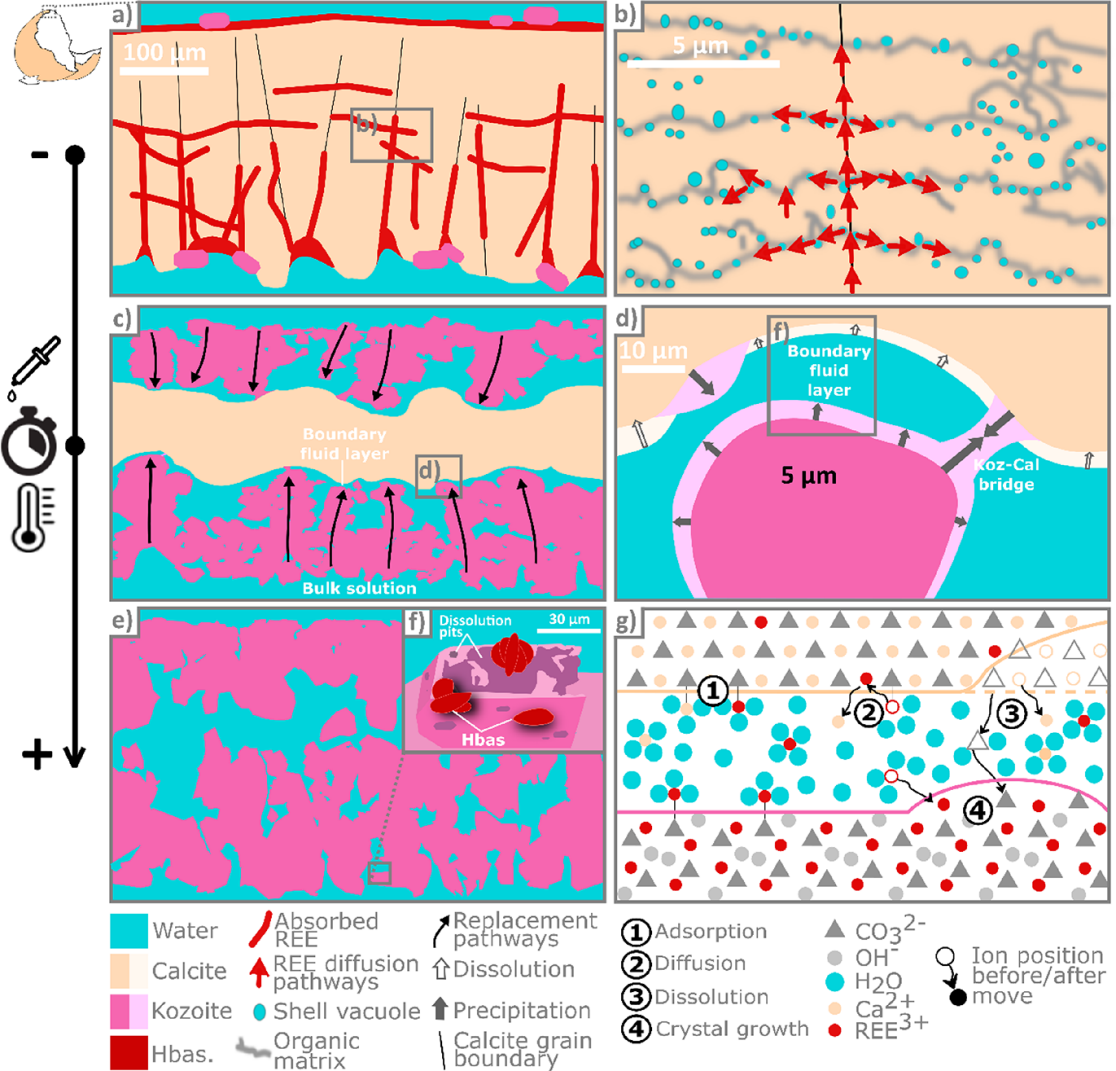
REE sorption by eggshell calcite. (a) REE diffusion within the grains, occurring at all temperatures; (b) the diffusion occurs primarily along the calcite crystal boundaries and the intracrystalline organic matrix; (c) at 165 and 205 °C, initiation of an interface-coupled dissolution–precipitation replacement of calcite by kozoite, along two centripetal replacement fronts; (d) the replacement occurs within a thin boundary fluid layer between the growing kozoite and dissolving calcite, with only minor kozoite precipitation directly on calcite; (e) after a few days, the calcite is entirely dissolved and replaced by porous pseudomorphic polycrystalline kozoite; (f) at 205 °C, after a few days, the kozoite starts to dissolve and “desert rose”-shaped hydroxylbastnäsite precipitate on the surface of the dissolving kozoite; (g) schematic representation of the atomic scale interactions between the calcite, kozoite, and solution along the boundary layer (not to scale).

The absorption of REE by eggshell calcite and the coupled dissolution–precipitation replacement represent two sorption mechanisms of REE by eggshell calcite, schematically illustrated in Figure 5.

### 3.5 REE Partitioning During Calcite Diffusion and Kozoite Replacement

LA-ICP-MS trace element mapping of the calcite grains after 95 days at 30 °C, 11 days at 90 °C, and 1 day at 165 °C shows that the absorbed La, Nd, and Dy were of equal concentrations (SI Figure S4). The identical concentration of all three REE, reflecting their identical concentration in the starting bulk solution, even after long reaction and absorption times, indicates that there is no significant REE partitioning that occurred during diffusion within the eggshell calcite.

**Figure 6:**
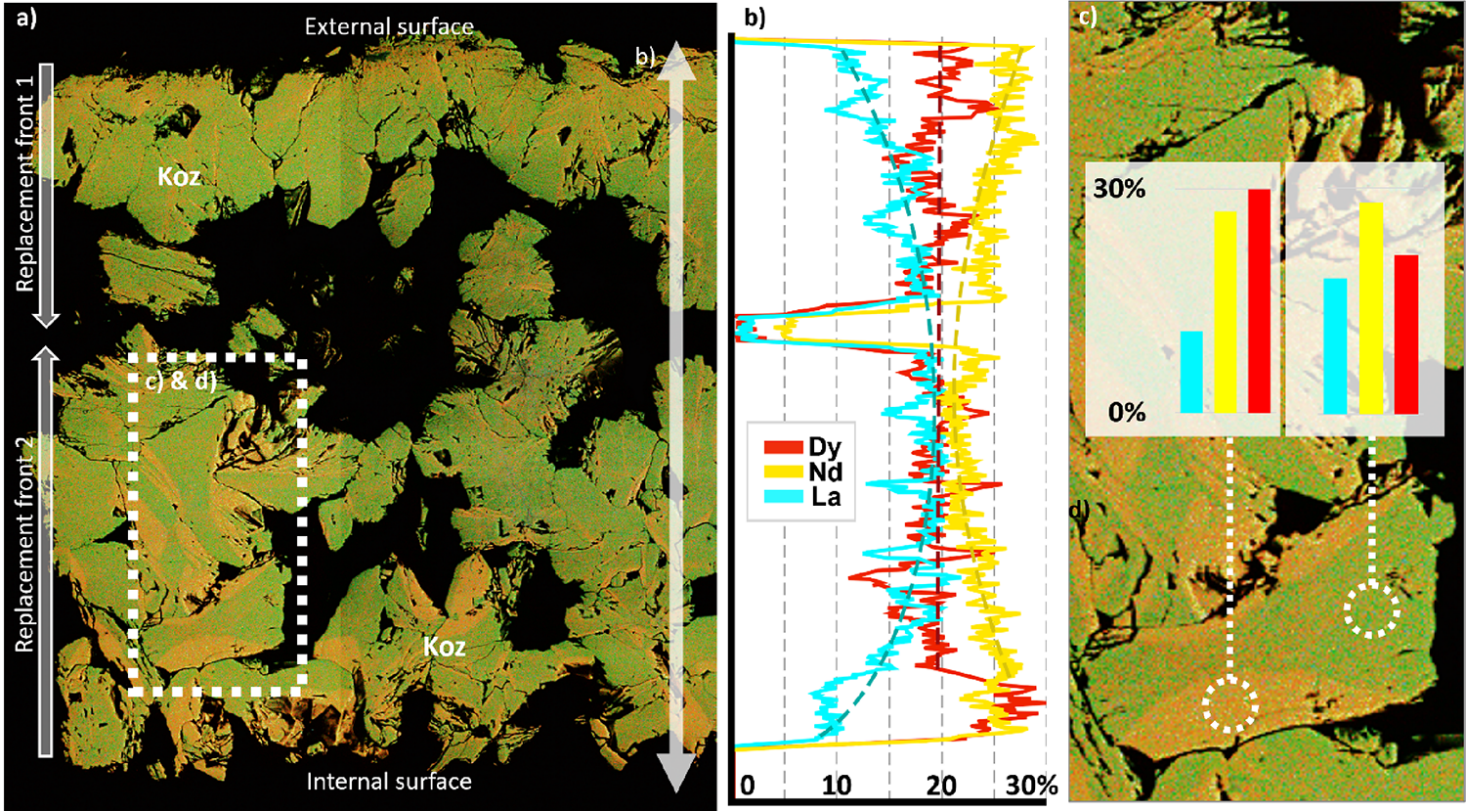
REE zoning in kozoite from eggshells at 165 °C after 11 days. (a) SEM-EDS map with La (blue), Nd (yellow), and Dy (red), underlain by a BSE image. (b) Quantitative EDS analysis along the eggshell cross section, showing centripetal zoning of La and Nd. (c) “Hourglass” zonation, with the greener zone showing equal amount of La and Dy, while the orange zone shows more Dy (+10% in comparison to the green zone) but less La (−10%).

On the contrary, the product of the coupled dissolution–precipitation reaction, kozoite, displays a heterogeneous spatial distribution of La, Nd, and Dy. EDS mapping shows that concentration of La increases from 10 to 20% from the grain rims to their cores, while Nd decreases from 25 to 20% from rims to cores, and Dy remains constant at ca. 20% (Figure 6a,b). Individual crystals of kozoite often show some zoning, visible on flat sections as repeating thin curvilinear features or, more rarely, as “hourglass” zoning (Figure 6a). A representative grain with hourglass zoning yielded ca. 20, 30, and 20% of La, Nd, and Dy in one zone, while the other zone yielded 10, 30, and 30% (Figure 6c), respectively, demonstrating limited partitioning of La and Dy.

Therefore, while the diffusion of REE within eggshell calcite does not partition the elements, the replacement of calcite by kozoite is accompanied by some partitioning at both the scale of the grain and the scale of individual kozoite crystals. The former is the most interesting from a beneficiation point of view, as millimeter-scale zoning might be more easily separated via comminution than μm-scale zoning. While a partitioning of ±10% is interesting to observe, it is not by itself sufficient to be exploited for industrial purpose. The magnitude of the partitioning process would have to be enhanced to yield higher partitioning coefficients.

## 4 Conclusions

The unique characteristic of eggshell calcite makes it a potential sustainable sorbent and precipitant for REE. The presence of an intracrystalline organic matrix and numerous grain boundaries results in the diffusion of REE along these features and their trapping (Figure 5a,b), enhancing the natural adsorption capacity of the calcite surface itself. The combination of recycling a waste product and being able to capture REE at room temperature, *i.e*., without any expenditure of energy for heating, makes the hen eggshell waste a low-cost and environmentally friendly sorbent, but only if the necessary long sorption time (weeks to months) is not an impediment (*e.g*., for passive treatment of polluted waters).

In addition, our study demonstrates that - at higher temperatures, eggshell calcite also has the potential to be used as a precipitant. On one hand, the intracrystalline organic matrix slows down the dissolution of calcite in acidic water, when compared to inorganic calcite. On the other hand, the low epitaxial match between eggshell calcite and kozoite, and the reduction in REE concentration within the boundary fluid layer due to localized absorption inside the grain, slows down the precipitation of kozoite. Both slowing down effects result in a slow interface coupled diffusion-precipitation reaction, allowing for the growth of large crystals of kozoite and the development of intracrust porosity (Figure 5c,d) and ultimately, the full dissolution of the calcite substrate and the production of rare earth carbonate pseudomorphs (Figure 5e–g). In the context of the use of calcite as a sacrificial substrate for an environmental or industrial application, full dissolution is a desired outcome as it maximizes the recycling of this waste product and simplifies the postreplacement beneficiation process by avoiding the presence of nonrare earth carbonate phases in the final product, as would be the case with remnants of inorganic calcite cores armored by kozoite. In this respect, eggshell calcite appears superior to inorganic calcite crystals. The dissolution–precipitation reaction also generates minor grain-scale REE partitioning, which, if magnified, could be exploited as an environmentally friendly technique for REE separation.

## Supporting Information Available

Calculation of the annual worldwide production of hen eggshells; pin stub and puck preparation; results of the quantitative analysis of the SEM secondary elemental standard; solubility of the rare earth hydroxycarbonates; compilation of published solubility product constants for kozoite and hydroxylbastnäsite; standard enthalpies of formation of the reactant and products in the REE hydroxycarbonate dissolution reaction; PHREEQC modeling rationales and input data; simplified representation of the hen eggshell calcite for the PHREEQC dissolution kinetic modeling; PHREEQC kinetic model input data for the modeling of the speed of calcite dissolution; PHREEQC input file; geochemical modeling results; binocular microscope photographs of the experiment products at 90, 165, and 205 °C; LA-ICP-MS maps of La, Nd, and Dy concentration in calcite (PDF)
