# Supplementary material for: Utilization of Eggshell Waste Calcite as a Sorbent for Rare Earth Element Recovery
Source: ACS Omega. 2024 Jun 4;9(24):25986–95. doi: 10.1021/acsomega.4c00931 (PMC11191108; doi:10.1021/acsomega.4c00931)
Supplement: Supplementary file 1 — ao4c00931_si_001.pdf [file ao4c00931_si_001.pdf]

Supporting Information For

Utilization of eggshell waste calcite as a sorbent for rare earth element recovery

Rémi Rateau[a], Melanie Maddin[a§], Adrienn M. Szucs[a†§], Luca Terribili[a§], Kerstin Drost[a,b], Paul C. Guyett[a,b], Juan Diego Rodriguez-Blanco[a,b]*

[a] Trinity College Dublin, the University of Dublin, Discipline of Geology, School of Natural Sciences, Museum Building, College Green, Dublin, D02 PN40, Ireland

[b] iCRAG, the Science Foundation Ireland Research Centre in Applied Geosciences, O'Brien Centre for Science (East), Dublin, Ireland

[*] rrateau@tcd.ie, +353 (0)89 499 6948

[§] These three authors contributed equally to this paper.

This supporting information contains 24 pages.

- SI Text S1-5
- SI Figure S1-4
- SI Table S1-4

1. **EXTENDED INTRODUCTION** 3

SI TEXT S1 - CALCULATION OF THE ANNUAL WORLDWIDE PRODUCTION OF HEN EGGSHELLS 3

2. **EXTENDED MATERIALS AND METHODS** 4

SI TEXT S2 – PIN STUBS AND PUCKS PREPARATION 4
SI TABLE S1 - RESULTS OF THE QUANTITATIVE ANALYSIS OF THE SEM SECONDARY ELEMENTAL STANDARD 5
SI TEXT S3 - SOLUBILITY OF THE RARE EARTH HYDROXYCARBONATES 5
SI TABLE S2 - COMPILATION OF PUBLISHED SOLUBILITY PRODUCT CONSTANTS FOR KOZOITE AND HYDROXYLBASTNÄSITE 7
SI TABLE S3 - STANDARD ENTHALPIES OF FORMATION OF THE REACTANT AND PRODUCTS IN THE REE HYDROXYCARBONATE DISSOLUTION REACTION. 9
SI TEXT S4 - PHREEQC MODELLING RATIONALES AND INPUT DATA 9
SI FIGURE S1 - SIMPLIFIED REPRESENTATION OF THE HEN EGGSHELL CALCITE FOR THE PHREEQC DISSOLUTION KINETIC MODELLING .. 12
SI TABLE S4 - PHREEQC KINETIC MODEL INPUT DATA FOR THE MODELLING OF THE SPEED OF CALCITE DISSOLUTION 13
SI TEXT S5 - PHREEQC INPUT FILE 13

3. **EXTENDED RESULTS AND DISCUSSIONS** 20

SI FIGURE S2 – GEOCHEMICAL MODELLING RESULTS 20
SI FIGURE S3 - BINOCULAR MICROSCOPE PHOTOGRAPHS OF THE EXPERIMENT PRODUCTS 21
SI FIGURE S4 – LA-ICP-MS MAPS OF LA, ND AND DY CONCENTRATION IN CALCITE 22

4. **REFERENCES** 23

1. Extended Introduction

SI Text S1 - Calculation of the annual worldwide production of hen eggshells

The FAOSTAT database (https://www.fao.org/faostat/en/#data/QCL), accessed on 10/01/2023, was filtered using the following criteria: 1) REGIONS = "World +(Total)"; 2) ITEMS = "Livestock primary" > "a) Hen eggs in shell, fresh" (this item provides two values: number of eggs produced and number of laying hens); and b) "Livestock primary" > "Meat of chicken, fresh or chilled"; 3) ELEMENTS = a) "Production quantity"; and b) "Producing animals/slaughtered"; 4) YEARS = "2022".

The number of eggs for consumption produced in 2021 is represented here by the variable S (S = 1.652×10^{12}). For each laying hen raised that year, we count two eggs having been produced, as only 50 % of chicks are female and therefore an equal number of male chicks must have been born and then immediately killed. Therefore, the number of eggshells associated with the raising of laying hens is twice the number of hens, represented here by the variable H (H = 2 x 7.827×10^{9}). Finally, we count one eggshell for each of the 75 billion chickens killed for meat in 2022, represented by the variable M (M = 7.520×10^{10}).

Finally, the total number of hen eggshells produced worldwide in 2022 by the food industry, represented here by the variable E, is derived using the following calculation: $E = S + 2H + M = 1.652 x 10^{12} + 2 x 7.827 x 10^{9} + 7.520 x 10^{10} = 1.743 x 10^{12}$.

2. Extended Materials and Methods

SI Text S2 – Pin stubs and pucks preparation

Several representative grains of each experiment were placed with a tweezer on a taped 12 mm wide metallic stub and either 1) carbon coated using a 208carbon carbon coater (Cressington) with 12 nm-thick of pure graphite carbon (TED PELLA, Inc.), for standard SEM-SE and BSE imaging and EDS elemental spectrum analyses and mapping; or 2) gold-coated using a 108auto sputter coater (Cressington; Au(-Pd), 8 nm-thick) for high resolution SEM SE imaging.

Several representative grains of each experiment were placed on a taped flat surface and inside a SeriForm 25 mm mounting cup (Struers) in which a mixture of EpoFix resin and hardener (Struers) was poured. The resin was left to solidify overnight in an oven at 50 ˚C. The resin pucks were then polished using a LaboPol-30 polishing machine fitted with a LaboForce-100 control panel/specimen mover unit (Struers) and a three-steps procedure using polishing fluids with 9, 6 and 1 μm diamond particles to expose internal surfaces of the grains. The 1 μm-polished pucks were cleaned in an ultrasonic bath of deionized water for three minutes. Isopropanol-based PELCO colloidal graphite (TED PELLA, Inc.) was painted on the side and circumference of the pucks.

The pucks were then carbon-coated as described with the stubs above. Pucks selected for LA-ICP-MS mapping were then cleaned with alcohol and re-polished at 1 μm for one minute and cleaned in an ultrasonic bath for three minutes before ablation.

SI Table S1 - Results of the quantitative analysis of the SEM secondary elemental standard

Table S1: SEM EDS quantitative analysis of the secondary elemental standard, a monazite from the MINM25-53 (serial 1CT) standard (Astimex Standards Ltd.); only the results for the relevant REE are presented.

**REE**	**Expected values (%)**	**Measured values (%)**	**Difference**
**La**	8.08	8.00	-1.0 %
**Nd**	9.43	9.45	0.2 %
**Dy**	0.80	0.77	-3.8 %

SI Text S3 - Solubility of the rare earth hydroxycarbonates

Existing data

The solubility product constants of kozoite and hydroxylbastnäsite has only been measured for a handful of pure phases where the lanthanide site is occupied by a single rare earth element (SI Table S2).

The majority of analyses have been dedicated to kozoite-(Nd), as Nd3+ can be used as a representative proxy for trivalent actinides in radioactive waste studies.[1] The latest experimental measurement of the solubility product constant of kozoite-(Nd), for the dissolution reaction $REE(CO_3)(OH)_{cr,orthorhombic} \rightleftharpoons Ln^{3+}_{aq} + OH^{-}_{aq} + CO_3^{2-}{}_{aq}$, yielded a value of $10^{-22.3 \pm 0.2}$ [2]; close to two orders of magnitude lower than the previous thermochemical calculation [3], illustrating the large uncertainties associated with the measurement or calculation of KSP. The only two other known kozoite K_{SP} are for kozoite-(Sm), at $10^{-21.2 \pm 0.7}$ [3], and kozoite-(Eu), at $10^{-20.3 \pm 1.0}$ [3] (SI Table S2).

The solubility product constant of hydroxylbastnäsite, for the dissolution reaction $Ln(CO_3)(OH)_{cr,hexagonal} \rightleftharpoons Ln^{3+}_{aq} + OH^{-}_{aq} + CO_3^{2-}{}_{aq}$, has only recently been investigated (SI Table S2). The K_{SP} of hydroxylbastnäsite-(La) was measured at $10^{-24.1 \pm 0.3}$ [2], one order of magnitude lower than the previous estimation using thermochemical calculation [3]. The K_{SP} of hydroxylbastnäsite-(Nd) was measured at $10^{-23.8 \pm 0.1}$ [2], in the error of the previous thermochemical

calculation [3]. The K_{SP} of hydroxylbastnäsite-(Sm) and –(Eu) was calculated to be, respectively, $10^{-24.2 \pm 0.6}$ and $10^{-24.2 \pm 0.6}$ [3] (SI Table S2).

For this study, kozoite-(Nd) and hydroxylbastnäsite-(Nd) will be used as representative phases for our synthetic rare earth hydroxycarbonate solid solutions, as 1) Nd chemical and physical properties are intermediate between the properties of La and Dy; and 2) there are no measurements of the solubility product constants of kozoite-(La), kozoite-(Dy), and hydroxylbastnäsite-(Dy) (SI Table S2).

For both kozoite-(Nd) and hydroxylbastnäsite-(Nd), we select the values from the latest study [2], as they are likely to be the most accurate and also can be compared to each other as they have been derived using the same protocol and assumptions.

SI Table S2 - Compilation of published solubility product constants for kozoite and hydroxylbastnäsite

Table S2: Compilation of logarithms of solubility product constants for kozoite and hydroxylbastnäsite.

Reference	La	Nd	Sm	Eu
Kozoite-(REE)				
4				-20.49
5		-19.19<br>-20.7*		
6		-19.94 ± 0.16<br>-21.4 ± 0.3*		-20.18 ± 0.16<br>-21.6 ± 0.2*
7		-22.54**		
8		20.12 ± 0.09<br>-21.6 ± 0.09*		
9		-21.7 ± 0.29*		
10		-21.5***		-21.8
11		-21.3 ± 0.7	-21 ± 0.9	
3		-20.7 ± 0.6#	-21.2 ± 0.7#	-20.3 ± 1.0#
2		-22.3 ± 0.2		
Hydroxylbastnäsite-(REE)				
3	-23.3 ± 0.6#	-23.7 ± 0.6#	-24.2 ± 0.6#	-23.3 ± 1.0#
2	-24.1 ± 0.3	-23.8 ± 0.1		

* Corrected by Rorif et al., 2005 to standard K_{SP} at zero ionic strength.
** As quoted by Spahiu & Bruno, 1995.
*** Not measured; selected by the authors based on previous studies.
# Thermochemical calculation.
Bold value = Selected K_{SP} used in this study.

Derivation of the enthalpy of reaction

The solubility product constants are usually calculated at 25 ˚C. Moreover, in PHREEQC, the log K_{SP} parameter for each dissolution reaction is required to have been calculated at 25 ˚C. The solubility product constant at other temperatures than 25 ˚C is calculated by PHREEQC based on one of two methods; one of them using the enthalpy of reaction at 25 ˚C and the Van't Hoff equation [12]:

$$\ln \frac{K_{T_2}}{K_{T_1}} = \frac{\Delta_r H^0}{R}\left[\frac{1}{T_1} - \frac{1}{T_2}\right]$$

With T_1 and T_2 the initial (25 ˚C) and final temperatures; K_{T1} and K_{T2} the solubility product constant of the solid phase at, respectively, temperature $T_{1,25°C}$ and T_2; $\Delta_r H^0$ is the standard enthalpy of reaction at 25 ˚C, and R is the ideal gas constant. Reorganizing the equation to find K_{T2}, we obtain:

$$K_{T_2} = K_{T_1} e^{\frac{\Delta_r H^0}{R}\left[\frac{1}{T_1} - \frac{1}{T_2}\right]}$$

The standard enthalpies of reaction, $\Delta_r H^0$ for both kozoite-(Nd) and hydroxylbastnäsite-(Nd) are not published, but can be calculated using Hess's Law, by subtracting the sum of the enthalpies of formation of the products, $\Delta_f H^0$, multiplied by their stoichiometric coefficients, from the sum of

the enthalpies of formation of the reactants, also multiplied by their own stochiometric coefficients.

In the case of the rare earth hydroxycarbonates, we have:

$$Nd(CO_3)(\mathrm{OH})_{\mathrm{cr,orthogonal}} \rightleftharpoons Nd^{3+}_{aq} + OH^{-}_{aq} + CO^{2-}_{3,aq}$$

$$\Delta_r H^0 = \left(1 \times \Delta_f H^0(Nd^{3+}_{aq}) + 1 \times \Delta_f H^0(OH^{-}_{aq}) + 1 \times \Delta_f H^0(CO^{2-}_{3,aq})\right) - \left(1 \times \Delta_f H^0(Nd(CO_3)(OH)_{cr})\right)$$

The standard enthalpies of formation of each species are provided in SI Table S3. Using Hess's Law, we calculate for kozoite-(Nd) and hydroxylbastnäsite-(Nd) an enthalpy of reaction of, respectively, 143.9 and 13.8 kJ.mol^{-1}.

SI Table S3 - Standard enthalpies of formation of the reactant and products in the REE hydroxycarbonate dissolution reaction.

Table S3: Standard enthalpies of formation of the reactant and product in the rare earth hydroxycarbonate dissolution reaction.

Species	$\Delta_f H^0$ (kJ.mol^{-1})	Reference
$Ln^{3+}_{(aq)}$	-694.8 ± 2.0	Table 8 in [13]
$OH^{-}_{(aq)}$	-229.9	[14]
$CO_3^{2-}{}_{(aq)}$	-676.3	[14]
Kozoite-Nd	-1744.9	Table 5 in [3]
Hydroxylbastnäsite-Nd	-1614.8	Table 5 in [3]

SI Text S4 - PHREEQC modelling rationales and input data

Starting solution

The 1 L 50 mM single rare earth bearing solutions were modelled using a one-step dissolution reaction (REACTION command) of 50 mM of REE salt in 1 kg of pure water. The assumed equilibrium with atmospheric air was modelled by equilibrating the solution with CO_2 (EQUILIBRIUM_PHASES command). For gases, the saturation index is defined as the logarithm of their partial pressure, *i.e.*, of their concentration in air. At the time of the experiments, in 2022-2023, the selected representative concentration is 4.172 ppm of CO_2 [15].

The 15 mL of starting solution in the air-tight reactor was modelled using a mixing (MIX command) of 0.005% of each of three 1 kg 50 mM single rare earth solution. Following the mixing, the solution is put into contact with a fixed 0.005 mL volume of air, representing the head space inside the Teflon reactor. This volume remains constant in any subsequent reactions applied to the solution inside the reactor. This 15 mL mixed REE solution is named the "starting solution" in the rest of this study.

Dissolution of hen eggshell calcite

In PHREEQC, the dissolution of a crystalline phase can be modelled either from a purely thermodynamic point of view, using the REACTION keyword; or else it can be modelled from a kinetic point of view, using a kinetic model and the KINETICS keyword.

For thermodynamic modelling only, the input data for the modelling of a dissolution reaction are the name and quantity (in mol) of a dissolving phase, and the number of dissolution steps. The thermodynamic information for calcite is already comprised within the llnl.dat database under the name "calcite", which is the one to be used for this model. As > 95% of the hen eggshell is made of calcite, with the rest being water and the organic matrix [16], we make the simplification that the entirety of the 0.05 g of dissolving eggshell clasts is made of calcite. With a calcite molar mass of 100.0869 $g.mol^{-1}$, the dissolution can be modelled with a dissolution of $5.0x10^{-4}$ mol of calcite.

For kinetics modelling, We use the llnl.dat database built-in calcite dissolution kinetics model [17,18]. The model requires the definition of an initial quantity of calcite, $n_{0,cal}$ (M0 in the PHREEQC Basic code block); the initial specific surface area of calcite, SSA (PARM(1) in PHREEQC), given in $cm^2.mol^{-1}$; and an exponent, i (PARM(2) in PHREEQC), to model the dependence of the rate of reaction on grain size. The model is valid for the temperature range 5 to 60 ˚C [17].

To calculate the specific surface area, we will assume that the dissolving eggshell calcite is represented by a flattened square rectangular cuboid (SI Figure S1). Given the known experimental mass of 0.05 g, and a thickness of 0.03 cm, representative of the 0.02-0.04 cm range of hen eggshell thicknesses of eggshell [19]; we can set the dimension of the square faces to 0.85 x 0.85 cm, which then yields a cuboid density $D(g.cm^{-3}) = \frac{m}{V} = \frac{0.05}{0.85^2 \times 0.03} = 2.3$, representative of the density of eggshell calcite in the range of 2.24-2.39 [20] (SI Figure S1). As presented in the previous section, the initial mass of mineral to dissolve can be represented by 4.995704x10^{-4} mol of calcite. For the specific surface area, we assume that the dissolution occurs solely on the two square surfaces, and not on the narrow sides, as observed in the experiments. Therefore, the total surface area is equal to twice 0.85^2, or 1.445 cm^{-2}, which when divided by the quantity of calcite, yields a specific surface area of 2,892 $cm^2.mol^{-1}$ (Table S4). As we assume that the dissolution occurs on the two opposite square surfaces only, as the dissolution proceeds, the surface area remains the same, and the dissolution rate is not affected by the decreasing quantity of calcite remaining. Therefore, we set the coefficient i to a very small value, *e.g.*, 0.0001, so that the surface area of calcite remains constant at *ca.* 1.28 cm^{-2} (Table S4):

$$A = SSA * n_{0,cal} * \left(\frac{n_{cal}}{n_{0,cal}}\right)^{i}$$

$$\lim_{i \to 0} A\ (cm^2) = SSA * n_{0,cal} * \lim_{i \to 0} \frac{n_{cal}}{n_{0,cal}} = SSA * n_{0,cal} * 1 = SSA * n_{0,cal} = 1.445$$

SI Figure S1 - Simplified representation of the hen eggshell calcite for the PHREEQC dissolution kinetic modelling

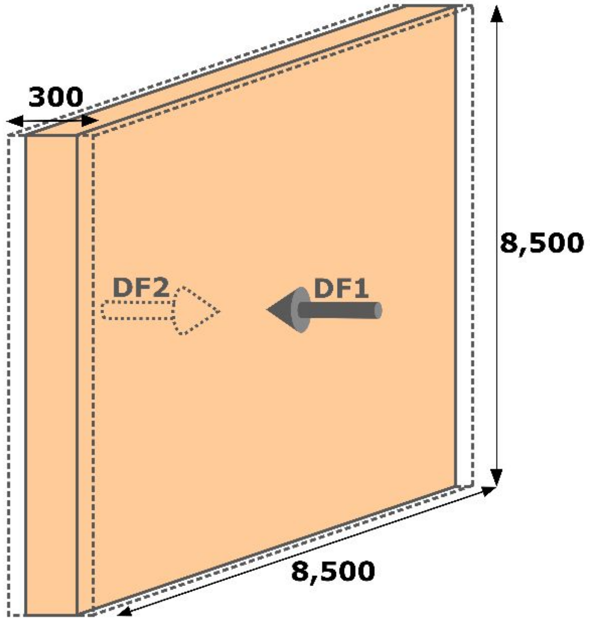

Figure S1. A simplified representation of the hen eggshell calcite for the PHREEQC dissolution kinetic modelling. DF1 = Direction of progression of planar dissolution front #1; DF2 = Direction of progression of planar dissolution front #2. All lengths are in μm.

SI Table S4 - PHREEQC kinetic model input data for the modelling of the speed of calcite dissolution

Table S4: PHREEQC kinetic model input data for the modelling of the speed of calcite dissolution. n = Amount of substance, SSA = Specific surface area, i = Coefficient for grain size-dependent dissolution rate.

	Unit	PHREEQC KINETICS block	PHREEQC Basic code	Value	Comment
$n_{0,cal}$	mol	-m0	M0	$5.0x10^{-4}$	0.05 g of calcite
n_{cal}	mol	-m	M	-	Default (= n_0)
**SSA**	$cm^2.mol^{-1}$	-parms [1]	PARM(1)	2892	=SA/n_0
**i**	Unitless	-parms [2]	PARM(2)	0.0001	For constant SA
**Tolerance**	mol	-tol	-	10^{-8}	Default
**Duration**	s	-steps	-	31557600	1 year

SI Text S5 - PHREEQC input file

```
########################################################
## EGGSHELL CALCITE + 50 mM MULTI-REE SOLUTION MODEL ##
########################################################

#######################
# LIST OF SIMULATIONS #
#######################
# (1) DISSOLUTION OF LANTHANUM SALT IN PURE WATER
# (2) DISSOLUTION OF NEODYMIUM SALT IN PURE WATER
# (3) DISSOLUTION OF DYSPROSIUM SALT IN PURE WATER
# (4) MODELLING OF THE STARTING SOLUTION FROM 25 TO 205 degC                              -> EXPORT
FILE (4)
# (5) MODELLING OF THE BULK DISSOLUTION OF 5 mg OF CALCITE BETWEEN 25 AND 205 degC        -> EXPORT
FILE (5)
# (6) MPDELLING OF THE STEPWISE DISSOLUTION OF 5 mg OF CALCITE AT A FIXED TEMPERATURE     -> EXPORT
FILE (6)
# (7) STEPWISE DISSOLUTION OF CALCITE @25degC, WITH KINETIC MODELLING                     -> EXPORT
FILE (7)
# (8) STEPWISE DISSOLUTION OF CALCITE @60degC, WITH KINETIC MODELLING                     -> EXPORT
FILE (8)

######################################################################
```

```
# THERMODYNAMIC DATABASE LLNL.DAT - USER-DEFINED ADDITIONAL  PHASES #
#####################################################################
PHASES

# KOZOITE-(ND)
Kozoite-(Nd)
      NdOHCO3  =  + 1.0000 Nd+++ + 1.0000 OH- + 1.0000 CO3--
      log_k           -22.3 # Voigt et al (2016) https://doi.org/10.1016/j.chemgeo.2016.03.012
      -delta_H        143.9 # kJ/mol, calculated using the enthalpies of formation
# Enthalpies of formation (kJ/mol):
#  NdOCCO3: -1744.9, from Rorif et al. (2005) https://doi.org/10.1016/j.chemgeo.2016.03.012
#  Nd+3: -694.8, from Rard (2016) https://doi.org/10.1007/s10953-016-0520-8
#  OH-: -229.9, from Sottery (1985) https://doi.org/10.1021/ed062pA325
#  CO3-2:-676.3, from Sottery (1985) https://doi.org/10.1021/ed062pA325
# Enthalpy of reaction = (1*-694.8 + 1*-229.9 + 1*-676.3) - (1*-1744.9) = 143.9 kJ/mol

# HYDROXYLBASTNASITE-(ND)
Hydroxylbastnasite-(Nd)
      NdOHCO3  =  + 1.0000 Nd+++ + 1.0000 OH- + 1.0000 CO3--
      log_k           -23.8 # Voigt et al (2016) https://doi.org/10.1016/j.chemgeo.2016.03.012
      -delta_H        13.8  # kJ/mol, calculated using the enthapies of formation
# Enthalpies of formation (kJ/mol):
#  NdOCCO3: -1614.8, from Rorif et al. (2005) https://doi.org/10.1016/j.chemgeo.2016.03.012
#  Nd+3: -694.8, from Rard (2016) https://doi.org/10.1007/s10953-016-0520-8
#  OH-: -229.9, from Sottery (1985) https://doi.org/10.1021/ed062pA325
#  CO3-2:-676.3, from Sottery (1985) https://doi.org/10.1021/ed062pA325
# Enthalpy of reaction = (1*-694.8 + 1*-229.9 + 1*-676.3) - (1*-1614.8) = 13.8 kJ/mol

#######################################################
TITLE (1) DISSOLUTION OF LANTHANUM SALT IN PURE WATER #
#######################################################
SOLUTION 1 Deoinized/pure water (1 L bottle), in equilibrium with air, at atmospheric conditions
    temp      25
    pH        7
    pe        4
    redox     pe
    units     mmol/kgw
    density      0.99705  #  from  "Wagner  &  Pruß  (1999)  The  IAPWS  Formulation  1995  [...]"
https://doi.org/10.1063/1.1461829 p.486
    -water    0.99705 # 1L @ 0.99705 density   = 0.99705 kg
REACTION 1 Dissolution of 50 mM of lanthanum nitrate salt (99.99%)
    La(NO3)3   1
    50 millimoles in 1 steps
EQUILIBRIUM_PHASES 1 Solution in equilibrium with CO2 in air
    CO2(g)     -3.3796557 10       # log10(0.0004172) from "Friedlingstein et al. (2022) Global
Carbon Budget 2022" https://doi.org/10.5194/essd-14-4811-2022
SAVE solution 1
END

#######################################################
TITLE (2) DISSOLUTION OF NEODYMIUM SALT IN PURE WATER #
#######################################################
SOLUTION 2 Deoinized/pure water (1 L bottle), in equilibrium with air, at atmospheric conditions
    temp      25
    pH        7.084
    pe        4
    redox     pe
    units     mmol/kgw
    density      0.99705  #  from  "Wagner  &  Pruß  (1999)  The  IAPWS  Formulation  1995  [...]"
https://doi.org/10.1063/1.1461829 p.486
    -water    0.99705 # 1L @ 0.99705 density   = 0.99705
REACTION 2 Neodymium nitrate salt (99.99%)
    Nd(NO3)3   1
    50 millimoles in 1 steps
EQUILIBRIUM_PHASES 2 In equlibrium with air
    CO2(g)     -3.3796557 10       # log10(0.0004172) from "Friedlingstein et al. (2022) Global
Carbon Budget 2022" https://doi.org/10.5194/essd-14-4811-2022
SAVE solution 2
END

########################################################
```

```
TITLE (3) DISSOLUTION OF DYSPROSIUM SALT IN PURE WATER #
#######################################################
SOLUTION 3 Deoinized/pure water (1 L bottle), in equilibrium with air, at atmospheric conditions
    temp      25
    pH        7.084
    pe        4
    redox     pe
    units     mmol/kgw
    density      0.99705  #  from  "Wagner  &  Pruß  (1999)  The  IAPWS  Formulation  1995  [...]"
https://doi.org/10.1063/1.1461829 p.486
    -water    0.99705 # 1L @ 0.99705 density   = 0.99705
REACTION 3 Dysprosium nitrate salt (99.99%)
    Dy(NO3)3   1
    50 millimoles in 1 steps
EQUILIBRIUM_PHASES 3 In equlibrium with air
    CO2(g)     -3.3796557 10       # log10(0.0004172) from "Friedlingstein et al. (2022) Global
Carbon Budget 2022" https://doi.org/10.5194/essd-14-4811-2022
SAVE solution 3
END

#################################################################
TITLE (4) MODELLING OF THE STARTING SOLUTION FROM 25 TO 205 degC #
#################################################################
MIX 1 Create starting solution by mixing 0.05 mL of solutions 1, 2 & 3
    1    0.005
    2    0.005
    3    0.005
GAS_PHASE 1 Starting solution in contact with fixed volume of air inside the Teflon reactor
    -fixed_volume
    -pressure 1
    -volume 0.005
    -temperature 25
    CO2(g)    0.0004172           # from "Friedlingstein et al. (2022) Global Carbon Budget 2022"
https://doi.org/10.5194/essd-14-4811-2022
# Reaction temperature, choose either #1 or #2 by commenting out the other one:
REACTION_TEMPERATURE 1 Heating of the starting solution, 1 degree increment between min and max
temperatures used for experiments
    25 205 in 181 steps
#REACTION_TEMPERATURE  1  Heating  of  the  starting  solution  at  the  temperatures  used  for  the
experiments
#    25 50 90 165 205
USER_GRAPH 1
    -chart_title                  "Starting solution"
    -headings                pH Mu
    -axis_scale x_axis     25 205 25 5
    -axis_titles             "Temperature (degC)" "pH" "Ionic strength"
    -initial_solutions       true
    -connect_simulations     true
  -start
10 PLOT_XY     TC, -LA("H+"), color = Blue, symbol = None, symbol_size = 8, y-axis = 1, line_width
= 4
20 PLOT_XY     TC, mu, color = Red, symbol = None, symbol_size = 8, y-axis = 2, line_width = 4
  -end
SELECTED_OUTPUT
        -file   (4)_STARTING SOLUTION_25-205_degC .pun
          -temperature
        -ionic_strength
          -ph
        -totals La(3) Nd(3) Dy(3)
        -molalities La+3 LaOH+2 LaCl+2 LaNO3+2 Nd+3 NdOH+2 NdCl+2 NdNO3+2 Dy+3 DyOH+2 DyCl+2 DyNO3+2
        -activities La+3 LaOH+2 LaCl+2 LaNO3+2 Nd+3 NdOH+2 NdCl+2 NdNO3+2 Dy+3 DyOH+2 DyCl+2 DyNO3+2
SAVE solution 4
END
USER_GRAPH 1
        -detach

############################################################################################
TITLE (5) (5) MODELLING OF THE BULK DISSOLUTION OF 5 mg OF CALCITE BETWEEN 25 AND 205 degC  #
############################################################################################
MIX 1 Create starting solution by mixing 0.05 mL of solutions 1, 2 & 3
    1    0.005
```

```
    2    0.005
    3    0.005
GAS_PHASE 1 Starting solution in contact with fixed volume of air inside the Teflon reactor
    -fixed_volume
    -pressure 1
    -volume 0.005
    -temperature 25
    CO2(g)    0.0004172           # from "Friedlingstein et al. (2022) Global Carbon Budget 2022"
https://doi.org/10.5194/essd-14-4811-2022
REACTION 4 Dissolve 500 umols of calcite
    Calcite 1
    500 micromoles in 1 steps
REACTION_TEMPERATURE 2
# Choose either 1) 180 steps over the entire range of experimental temperatures; or 2) the 4
experimental temperatures only
    25 205 in 180 steps
    #25 90 165 205
USER_GRAPH 2
    -chart_title                  "Calcite dissolution"
    -headings               SI(Cal) SI(Koz-Nd) SI(Koz-Hbas) pH
    -axis_scale x_axis     25 205 50 5
    -axis_titles            "Temperature, degC" "Saturation index" "pH"
    -initial_solutions      true
    -connect_simulations    true
  -start
10 PLOT_XY     TC, SI("Calcite") , color = Orange, symbol = None, symbol_size = 8, y-axis = 1,
line_width = 4
20 PLOT_XY     TC, SI("Kozoite-(Nd)") , color = Magenta, symbol = None, symbol_size = 8, y-axis =
1, line_width = 4
30 PLOT_XY     TC, SI("Hydroxylbastnasite-(Nd)") , color = Red, symbol = None, symbol_size = 8, y-
axis = 1, line_width = 4
40 PLOT_XY     TC, -LA("H+") , color = Black, symbol = None, symbol_size = 8, y-axis = 2, line_width
= 4
  -end
SAVE solution 4
SELECTED_OUTPUT
        -file   (5)_CALCITE_BULK_DISSOLUTION_VARYING_TEMPERATURES.pun
          -temperature
        -ionic_strength
          -ph
        -totals Ca La(3) Nd(3) Dy(3)
        -molalities Ca La+3 LaOH+2 LaCl+2 LaNO3+2 Nd+3 NdOH+2 NdCl+2 NdNO3+2 Dy+3 DyOH+2 DyCl+2
DyNO3+2
        -activities Ca La+3 LaOH+2 LaCl+2 LaNO3+2 Nd+3 NdOH+2 NdCl+2 NdNO3+2 Dy+3 DyOH+2 DyCl+2
DyNO3+2
        -saturation_indices Calcite Kozoite-(Nd) Hydroxylbastnasite-(Nd)
END
USER_GRAPH 2
        -detach

###########################################################################################
TITLE (6) MPDELLING OF THE STEPWISE DISSOLUTION OF 5 mg OF CALCITE AT A FIXED TEMPERATURE #
###########################################################################################
TITLE Modelling of the stepwise dissolution of 5 mg of calcite at a fixed temperature
MIX 1 Create starting solution by mixing 0.05 mL of solutions 1, 2 & 3
    1    0.005
    2    0.005
    3    0.005
GAS_PHASE 1 Starting solution in contact with fixed volume of air inside the Teflon reactor
    -fixed_volume
    -pressure 1
    -volume 0.005
    -temperature 25
    CO2(g)    0.0004172           # from "Friedlingstein et al. (2022) Global Carbon Budget 2022"
https://doi.org/10.5194/essd-14-4811-2022
REACTION 5 Dissolve 500 umols of calcite
    Calcite 1
    500 micromoles in 1000 steps
REACTION_TEMPERATURE 3
# Choose either 25, 90, 165 or 205 (degC)
    165
```

```
USER_GRAPH 3
    -chart_title                  "Calcite dissolution"
    -headings                SI(Cal) SI(Koz-Nd) SI(Koz-Hbas) pH
    -axis_scale x_axis     0 500 50 5
    -axis_titles             "[Ca], umol" "Saturation index" "pH"
    -initial_solutions       true
    -connect_simulations     true
  -start
10 PLOT_XY     TOT("Ca")*0.015*1000000, SI("Calcite") , color = Orange, symbol = None, symbol_size = 8, y-axis = 1, line_width = 4
20 PLOT_XY     TOT("Ca")*0.015*1000000,  SI("Kozoite-(Nd)")  ,  color  =  Magenta,  symbol  =  None, symbol_size = 8, y-axis = 1, line_width = 4
30 PLOT_XY     TOT("Ca")*0.015*1000000, SI("Hydroxylbastnasite-(Nd)") , color = Red, symbol = None, symbol_size = 8, y-axis = 1, line_width = 4
40 PLOT_XY     TOT("Ca")*0.015*1000000, -LA("H+") , color = Black, symbol = None, symbol_size = 8, y-axis = 2, line_width = 4
  -end
SELECTED_OUTPUT
        -file   (6)_CALCITE_STEPWISE_DISSOLUTION_FIXED_TEMPERATURE.pun
         -temperature
        -ionic_strength
         -ph
        -totals Ca La(3) Nd(3) Dy(3)
        -molalities Ca La+3 LaOH+2 LaCl+2 LaNO3+2 Nd+3 NdOH+2 NdCl+2 NdNO3+2 Dy+3 DyOH+2 DyCl+2 DyNO3+2
        -activities Ca La+3 LaOH+2 LaCl+2 LaNO3+2 Nd+3 NdOH+2 NdCl+2 NdNO3+2 Dy+3 DyOH+2 DyCl+2 DyNO3+2
        -saturation_indices Calcite Kozoite-(Nd) Hydroxylbastnasite-(Nd)
END
USER_GRAPH 3
        -detach

###########################################################################
TITLE (7) STEPWISE DISSOLUTION OF CALCITE @25degC, WITH KINETIC MODELLING #
###########################################################################
MIX 1 Create starting solution by mixing 0.05 mL of solutions 1, 2 & 3
    1    0.005
    2    0.005
    3    0.005
GAS_PHASE 1 Starting solution in contact with fixed volume of air inside the Teflon reactor
    -fixed_volume
    -pressure 1
    -volume 0.005
    -temperature 25
    CO2(g)    0.0004172           # from "Friedlingstein et al. (2022) Global Carbon Budget 2022" https://doi.org/10.5194/essd-14-4811-2022
REACTION_TEMPERATURE 1 Heat starting solution
    25
INCREMENTAL_REACTIONS true
KINETICS 1 Incremental dissolution of calcite until saturation is reached SI(calcite)=0
# Default kinetic model for calcite as defined in the LLNL database, from Plummer et al. (1978), Plummer et al. (?) AJS 278, 179; , Appelo et al. (?) Appelo et al., AG 13, 257.
# In mmol/cm2/s
# Valid for temperature range of 5-60 degC
# KINETICS input data manual: https://wwwbrr.cr.usgs.gov/projects/GWC_coupled/phreeqc/html/final-45.html
Calcite
    -formula  CaCO3  1
    #-m        0.00050   # moles, default equal to m0
    -m0       0.00050    # moles
    -parms    2890 0.0001     # cm^2/mol calcite, exp factor to keep active surface area constant
    -tol      1e-08
-steps       31557600 in 100 steps # 31557600 seconds = 1 year
-step_divide 1
-runge_kutta 3
-bad_step_max 500
USER_GRAPH 4
    -chart_title                  "Quantity of dissolved calcite vs SI and pH @25degC"
    -headings                SI(Cal)@25degC SI(Koz-Nd)@25degC SI(Hbas-Nd)@25degC pH@25degC
    -axis_scale x_axis     0 500 50 10
    -axis_titles             "[Ca], umol" "Saturation index" "pH"
```

```
    -initial_solutions      true
    -connect_simulations    false
  -start
10 PLOT_XY     TOT("Ca")*0.015*1000000, SI("Calcite") , color = Orange, symbol = None, symbol_size
= 8, y-axis = 1, line_width = 2.5
20 PLOT_XY     TOT("Ca")*0.015*1000000,  SI("Kozoite-(Nd)")  ,  color  =  Magenta,  symbol  =  None,
symbol_size = 8, y-axis = 1, line_width = 2.5
30 PLOT_XY     TOT("Ca")*0.015*1000000, SI("Hydroxylbastnasite-(Nd)") , color = Red, symbol = None,
symbol_size = 8, y-axis = 1, line_width = 2.5
40 PLOT_XY     TOT("Ca")*0.015*1000000, -LA("H+") , color = Black, symbol = None, symbol_size = 8,
y-axis = 2, line_width = 2.5
  -end
USER_GRAPH 5
    -chart_title                  "Quantity of dissolved calcite vs calcite SI and dissolution time
@50degC"
    -headings               SI(cal)@25degC Dissolution_time@25degC
    -axis_titles            "[Calcite], umol" "SI (cal)" "Time (days)
    -axis_scale x_axis     0 500 50 10
    -initial_solutions      true
    -connect_simulations    false
  -start
10 PLOT_XY     TOT("Ca")*0.015*1000000, SI("Calcite"), color = Orange, symbol = None, symbol_size
= 8, y-axis = 1, line_width = 2.5
20 PLOT_XY     TOT("Ca")*0.015*1000000, TOTAL_TIME/86400, color = Black, symbol = None, symbol_size
= 8, y-axis = 2, line_width = 2.5
  -end
SELECTED_OUTPUT
        -file   (7)_CALCITE_STEPWISE_DISSOLUTION_KINETOCS_AT_25degC.pun
          -temperature
        -ionic_strength#
          -ph
        -saturation_indices Calcite
        -totals Ca La(3) Nd(3) Dy(3)
        -molalities La+3 LaOH+2 LaCl+2 LaNO3+2 Nd+3 NdOH+2 NdCl+2 NdNO3+2 Dy+3 DyOH+2 DyCl+2 DyNO3+2
        -activities La+3 LaOH+2 LaCl+2 LaNO3+2 Nd+3 NdOH+2 NdCl+2 NdNO3+2 Dy+3 DyOH+2 DyCl+2 DyNO3+2
END

###########################################################################
TITLE (8) STEPWISE DISSOLUTION OF CALCITE @60degC, WITH KINETIC MODELLING #
###########################################################################
MIX 1 Create starting solution by mixing 0.05 mL of solutions 1, 2 & 3
    1    0.005
    2    0.005
    3    0.005
GAS_PHASE 1 Starting solution in contact with fixed volume of air inside the Teflon reactor
    -fixed_volume
    -pressure 1
    -volume 0.005
    -temperature 25
    CO2(g)    0.0004172          # from "Friedlingstein et al. (2022) Global Carbon Budget 2022"
https://doi.org/10.5194/essd-14-4811-2022
REACTION_TEMPERATURE 1 Heat starting solution
# Select any temperature between 25 and 60 degC
    60
INCREMENTAL_REACTIONS true
KINETICS 1 Incremental dissolution of calcite until saturation is reached SI(calcite)=0
# Default kinetic model for calcite as defined in the LLNL database, from Plummer et al. (1978),
Plummer et al. (?) AJS 278, 179; , Appelo et al. (?) Appelo et al., AG 13, 257.
# In mmol/cm2/s
# Valid for temperature range of 5-60 degC
# KINETICS input data manual: https://wwwbrr.cr.usgs.gov/projects/GWC_coupled/phreeqc/html/final-
45.html
Calcite
    -formula  CaCO3  1
    #-m        0.0004995704   # moles, default is m0
    -m0       0.0004995704    # moles
    -parms    2890 0.0001     # cm^2/mol calcite, exp factor to keep active surface area constant
    -tol      1e-08
-steps       31557600 in 1001 steps # 31557600 seconds = 1 year
-step_divide 1
-runge_kutta 3
```

```
-bad_step_max 500
USER_GRAPH 4
    -chart_title                  "Quantity of dissolved calcite vs SI and pH @25degC"
    -headings               SI(Cal)@50degC SI(Koz-Nd)@50degC SI(Hbas-Nd)@50degC pH@50degC
    -axis_scale x_axis     0 500 50 10
    -axis_titles            "[Ca], umol" "Saturation index" "pH"
    -initial_solutions      true
    -connect_simulations    false
  -start
10 PLOT_XY     TOT("Ca")*0.015*1000000, SI("Calcite") , color = Orange, symbol = None, symbol_size
= 8, y-axis = 1, line_width = 5
20 PLOT_XY     TOT("Ca")*0.015*1000000,  SI("Kozoite-(Nd)")  ,  color  =  Magenta,  symbol  =  None,
symbol_size = 8, y-axis = 1, line_width = 5
30 PLOT_XY     TOT("Ca")*0.015*1000000, SI("Hydroxylbastnasite-(Nd)") , color = Red, symbol = None,
symbol_size = 8, y-axis = 1, line_width = 5
40 PLOT_XY     TOT("Ca")*0.015*1000000, -LA("H+") , color = Black, symbol = None, symbol_size = 8,
y-axis = 2, line_width = 5
  -end
USER_GRAPH 5
    -chart_title                  "Quantity of dissolved calcite vs calcite SI and dissolution time
@50degC"
    -headings               SI(cal)@50degC Dissolution_time@50degC
    -axis_titles            "[Calcite], umol" "SI (cal)" "Time (days)
    -axis_scale x_axis     0 500 50 10
    -initial_solutions      true
    -connect_simulations    false
  -start
10 PLOT_XY     TOT("Ca")*0.015*1000000, SI("Calcite"), color = Orange, symbol = None, symbol_size
= 8, y-axis = 1, line_width = 5
20 PLOT_XY     TOT("Ca")*0.015*1000000, TOTAL_TIME/86400, color = Black, symbol = None, symbol_size
= 8, y-axis = 2, line_width = 5
  -end
SELECTED_OUTPUT
        -file   (8_CALCITE_STEPWISE_DISSOLUTION_KINETOCS_AT_60degC.pun
         -temperature
        -ionic_strength#
         -ph
        -saturation_indices Calcite
        -totals Ca La(3) Nd(3) Dy(3)
        -molalities La+3 LaOH+2 LaCl+2 LaNO3+2 Nd+3 NdOH+2 NdCl+2 NdNO3+2 Dy+3 DyOH+2 DyCl+2 DyNO3+2
        -activities La+3 LaOH+2 LaCl+2 LaNO3+2 Nd+3 NdOH+2 NdCl+2 NdNO3+2 Dy+3 DyOH+2 DyCl+2 DyNO3+2
END
USER_GRAPH 4
        -detach
USER_GRAPH 5
        -detach
```

3. Extended Results and Discussions

SI Figure S2 – Geochemical modelling results

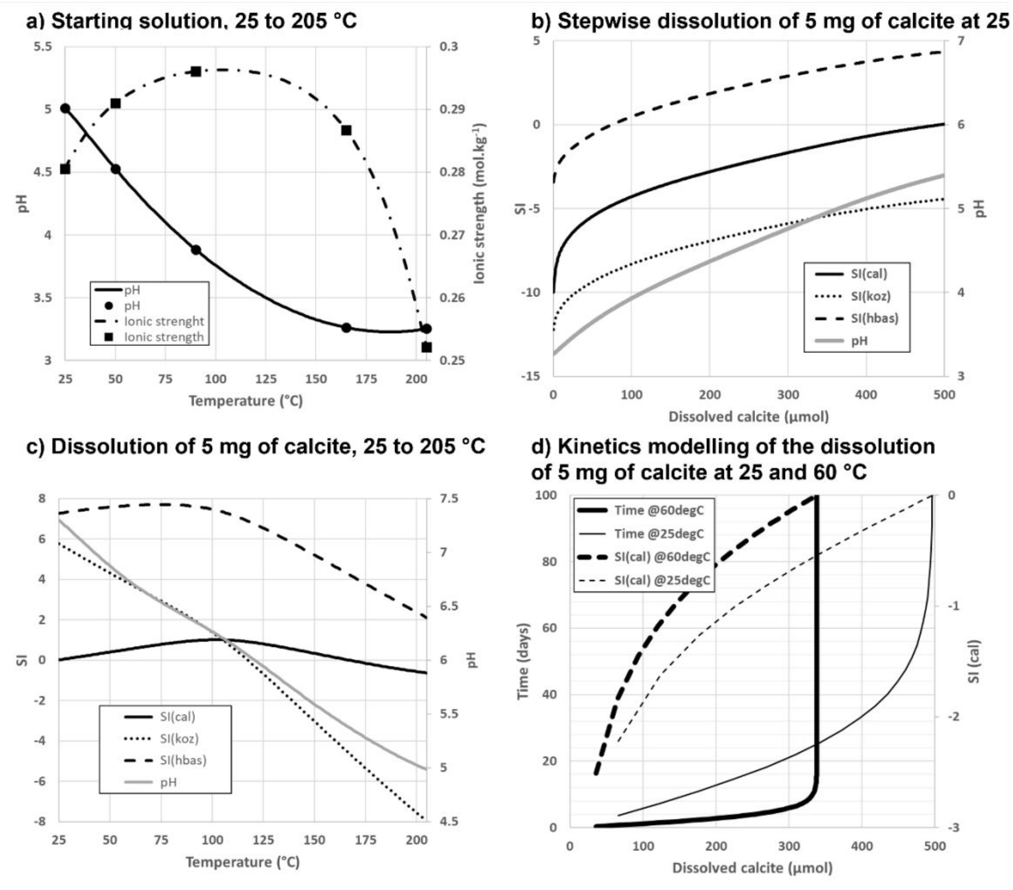

Figure S2: Selected results of the geochemical modelling of the starting solution and experiments. a) Starting solution, temperature *vs* pH (left y-axis) and vs ionic strength (right y-axis); b) Stepwise dissolution of 5 mg of calcite at 25 ˚C. Mass of dissolved calcite *vs* saturation indices of calcite, kozoite and hydroxylbastnäsite (left y-axis), and vs pH (right y-axis); c) Dissolution of 5 mg of calcite between 25 and 205 ˚C. Temperature vs saturation indices (left y-axis), and vs pH (right y-

axis); and d) Kinetics modelling of the dissolution of 5 mg of calcite at 25 ˚C (thin black lines) and at 60 ˚C (thick black lines). Mass of dissolved calcite vs time (in days, left y-axis), and vs saturation index of calcite (right y-axis).

SI Figure S3 - Binocular microscope photographs of the experiment products

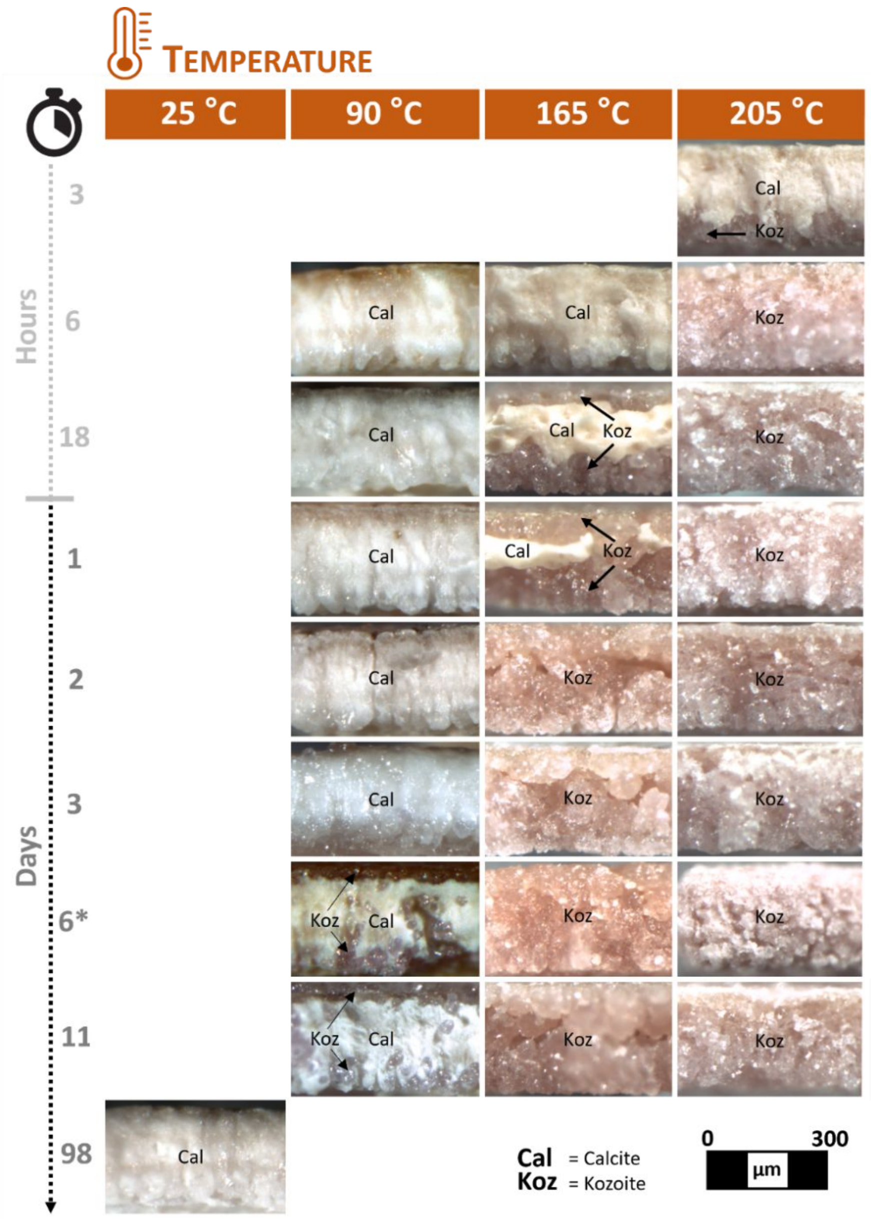

Figure S3: Binocular microscope photographs of the experiment products at 25, 90, 165 and 205 ˚C. All photos show the eggshell cross-section, with the external surface at the top and the internal surface at the bottom. The kozoite crystals have a pink hue, indicating the incorporation of Nd. Note the progression of the two replacement fronts in the first four photographs at 165 ˚C. *: 6±1 days; 7 days at 90 and 205˚C, 5 days for the 165˚C experiment.

SI Figure S4 – LA-ICP-MS maps of La, Nd and Dy concentration in calcite

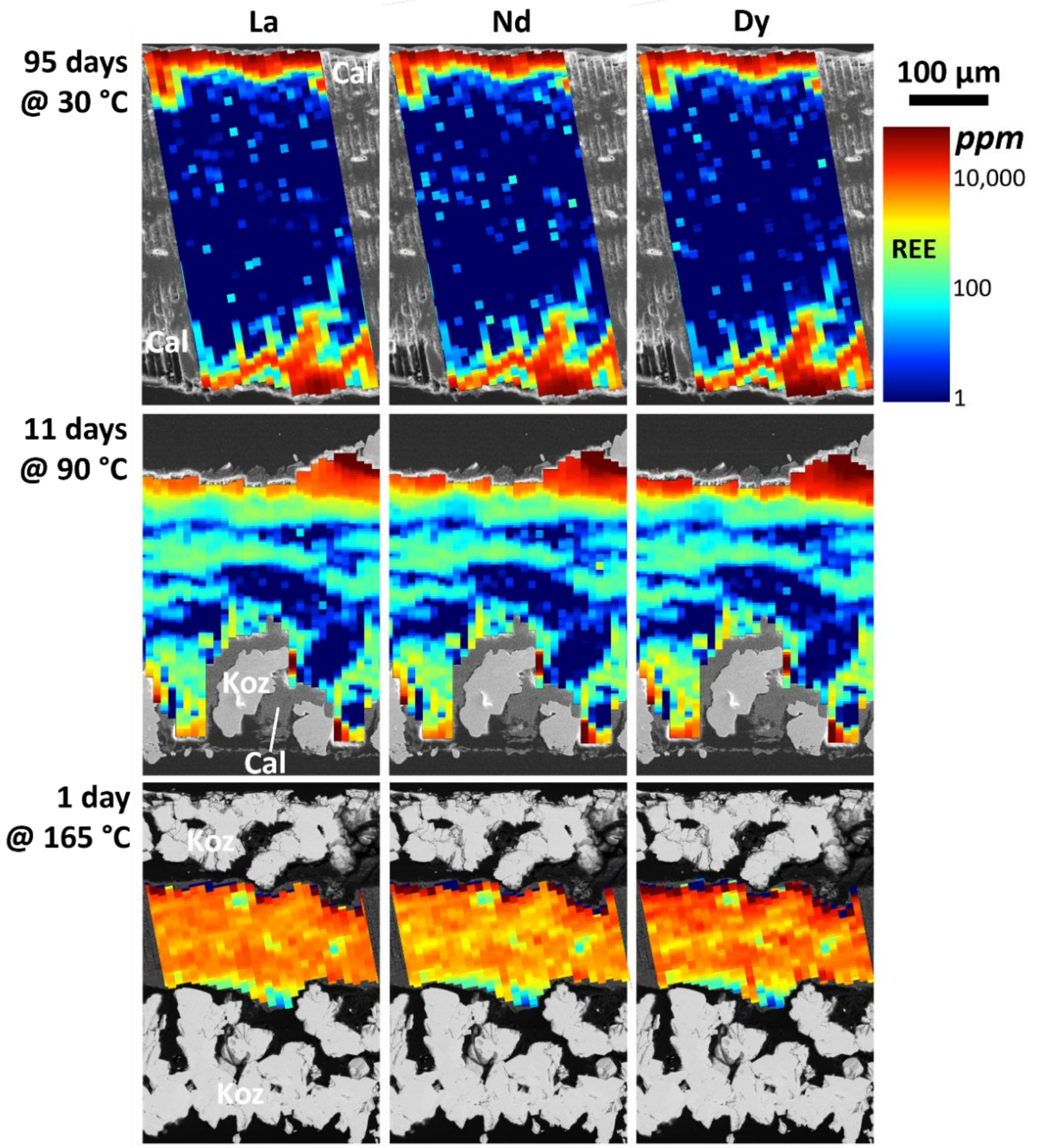

Figure S4: Maps of concentration of La, Nd and Dy for selected experiments at 30, 90 and 165 ˚C. For all experiments, the concentrations of all three REE is visually equal, with no indication of any significant partitioning between them. The kozoite crystals were not ablated by the laser as the ICP-MS was tuned for trace element concentration of REE and the ablation of minerals with 30+ % of REE could damage the electron multiplier detector of the mass spectrometer.

4. REFERENCES

(1) Brookins, D. G. *Geochemical Aspects of Radioactive Waste Disposal*; Springer: New York, NY, 1984. https://doi.org/10.1007/978-1-4613-8254-6.

(2) Voigt, M.; Rodriguez-Blanco, J. D.; Vallina, B.; Benning, L. G.; Oelkers, E. H. An Experimental Study of Hydroxylbastnasite Solubility in Aqueous Solutions at 25˚C. *Chemical Geology* **2016**, *430*, 70–77. https://doi.org/10.1016/j.chemgeo.2016.03.012.

(3) Rorif, F.; Fuger, J.; Desreux, J. F. Thermochemistry of Selected Trivalent Lanthanide and Americium Compounds: Orthorhombic and Hexagonal Hydroxycarbonates. *Radiochimica Acta* **2005**, *93* (2), 103–110. https://doi.org/10.1524/ract.93.2.103.59419.

(4) Bernkopf, M. F. Hydrolysereaktionen und karbonatkomplexierung von dreiwertigem Americium in naturlichen aquatischen System. PhD thesis, Institut fur Radiochemie der Technischen Universitat Munchen., 1984.

(5) Meinrath, G.; Kim, J. I. Solubility Products of Different Am (III) and Nd (III) Carbonates; European Journal of Solid State and Inorganic Chemistry: Leuven, Belgium, 1991; Vol. 28:Suppl.

(6) Runde, W.; Meinrath, G.; Kim, J. I. A Study of Solid-Liquid Phase Equilibria of Trivalent Lanthanide and Actinide Ions in Carbonate Systems. *Radiochimica Acta* **1992**, *58–59* (1), 93–100. https://doi.org/10.1524/ract.1992.5859.1.93.

(7) Rai, D.; Felmy, A. R.; Fulton, R. W.; Ryan, J. L. Aqueous Chemistry of Nd in Borosilicate-Glass/Water Systems. *Radiochimica Acta* **1992**, *58–59* (1), 9–16. https://doi.org/10.1524/ract.1992.5859.1.9.

(8) Meinrath, G.; Takeishi, H. Solid-Liquid Equilibria of Nd3+ in Carbonate Solutions. *Journal of Alloys and Compounds* **1993**, *194* (1), 93–99. https://doi.org/10.1016/0925-8388(93)90651-3.

(9) Carroll, S. A. Precipitation of Nd-Ca Carbonate Solid Solution at 25˚C. *Geochimica et Cosmochimica Acta* **1993**, *57* (14), 3383–3393. https://doi.org/10.1016/0016-7037(93)90546-9.

(10) Spahiu, K.; Bruno, J. *A Selected Thermodynamic Database for REE to Be Used in HLNW Performance Assessment Exercises*; 0284–3757; Sweden, 1995; p 90. http://inis.iaea.org/search/search.aspx?orig_q=RN:28019633.

(11) Merli, L.; Fuger, J. Thermochemistry of Selected Lanthanide and Actinide Hydroxycarbonates and Carbonates. *Radiochimica Acta* **1996**, *74* (s1), 37–44. https://doi.org/10.1524/ract.1996.74.special-issue.37.

(12) Parkhurst, D. L.; Appelo, C. a. J. *Description of Input and Examples for PHREEQC Version 3: A Computer Program for Speciation, Batch-Reaction, One-Dimensional Transport, and Inverse Geochemical Calculations*; 6-A43; U.S. Geological Survey, 2013. https://doi.org/10.3133/tm6A43.

(13) Rard, J. A. Critical Evaluation of the Standard Molar Entropies, Enthalpies of Formation, Gibbs Energies of Formation and Heat Capacities of the Aqueous Trivalent Rare Earth Ions, and the Corresponding Standard Molar Entropies, Enthalpies of Formation and Gibbs Energies of Formation of the Thermodynamically Stable RECl3·7H2O(Cr) and RECl3·6H2O(Cr). *J Solution Chem* **2016**, *45* (9), 1332–1376. https://doi.org/10.1007/s10953-016-0520-8.

(14) Sottery, T. W. Chemical Principles, Sixth Edition (Masterton, William L.; Slowinski, Emil J.; Stanitski, Conrad L.). *J. Chem. Educ.* **1985**, *62* (12), A325. https://doi.org/10.1021/ed062pA325.

(15) Friedlingstein, P.; O'Sullivan, M.; Jones, M. W.; Andrew, R. M.; Gregor, L.; Hauck, J.; Le Quéré, C.; Luijkx, I. T.; Olsen, A.; Peters, G. P.; Peters, W.; Pongratz, J.; Schwingshackl, C.; Sitch, S.; Canadell, J. G.; Ciais, P.; Jackson, R. B.; Alin, S. R.; Alkama, R.; Arneth, A.; Arora, V. K.; Bates, N. R.; Becker, M.; Bellouin, N.; Bittig, H. C.; Bopp, L.; Chevallier, F.; Chini, L. P.; Cronin, M.; Evans, W.; Falk, S.; Feely, R. A.; Gasser, T.; Gehlen, M.; Gkritzalis, T.; Gloege, L.; Grassi, G.; Gruber, N.; Gürses, Ö.; Harris, I.; Hefner, M.; Houghton, R. A.; Hurtt, G. C.; Iida, Y.; Ilyina, T.; Jain, A. K.; Jersild, A.; Kadono, K.; Kato, E.; Kennedy, D.; Klein Goldewijk, K.; Knauer, J.; Korsbakken, J. I.; Landschützer, P.; Lefèvre, N.; Lindsay, K.; Liu, J.; Liu, Z.; Marland, G.; Mayot, N.; McGrath, M. J.; Metzl, N.; Monacci, N. M.; Munro, D. R.; Nakaoka, S.-I.; Niwa, Y.; O'Brien, K.; Ono, T.; Palmer, P. I.; Pan, N.; Pierrot, D.; Pocock, K.; Poulter, B.; Resplandy, L.; Robertson, E.; Rödenbeck, C.; Rodriguez, C.; Rosan, T. M.; Schwinger, J.; Séférian, R.; Shutler, J. D.; Skjelvan, I.; Steinhoff, T.; Sun, Q.; Sutton, A. J.; Sweeney, C.; Takao, S.; Tanhua, T.; Tans, P. P.; Tian, X.; Tian, H.; Tilbrook, B.; Tsujino, H.; Tubiello, F.; van der Werf, G. R.; Walker, A. P.; Wanninkhof, R.; Whitehead, C.; Willstrand Wranne, A.; Wright, R.; Yuan, W.; Yue, C.; Yue, X.; Zaehle, S.; Zeng, J.; Zheng,

B. Global Carbon Budget 2022. *Earth System Science Data* **2022**, *14* (11), 4811–4900. https://doi.org/10.5194/essd-14-4811-2022.

(16) Nys, Y.; Guyot, N. Egg Formation and Chemistry. In *Improving the Safety and Quality of Eggs and egg Products*; 2011; pp 83–132. https://doi.org/10.1533/9780857093912.2.83.

(17) Plummer, L.; Wigley, T.; Parkhurst, D. The Kinetics of Calcite Dissolution in CO2-Water Systems at 5˚C to 60˚C and 0.0 to 1.0 Atm CO2. *American Journal of Science* **1978**, *278*, 179–216. https://doi.org/10.2475/ajs.278.2.179.

(18) Appelo, C. A. J.; Verweij, E.; Schäfer, H. A Hydrogeochemical Transport Model for an Oxidation Experiment with Pyrite/Calcite/Exchangers/Organic Matter Containing Sand. *Applied Geochemistry* **1998**, *13* (2), 257–268. https://doi.org/10.1016/S0883-2927(97)00070-X.

(19) Ketta, M.; Tůmová, E. Relationship between Eggshell Thickness and Other Eggshell Measurements in Eggs from Litter and Cages. *Italian Journal of Animal Science* **2018**, *17* (1), 234–239. https://doi.org/10.1080/1828051X.2017.1344935.

(20) Carter, T. C. The Hen's Egg: Density of Egg Shell and Egg Contents. *British Poultry Science* **1968**, *9* (3), 265–271. https://doi.org/10.1080/00071666808415718.
